# Adalimumab Safety in Inflammatory Bowel Disease: A Clinical Cohort Study with Time-to-Event Analysis and FAERS Signal Assessment

**DOI:** 10.3390/ph19081239

**Published:** 2026-08-06

**Authors:** Bojana Milašinović, Srđan Marković, Sandra Vezmar Kovačević, Petar Svorcan, Branislava Miljković, Katarina Vučićević

**Affiliations:** 1 Department of Pharmacokinetics and Clinical Pharmacy, Faculty of Pharmacy, University of Belgrade, 11000 Belgrade, Serbia; 2 Department of Gastroenterology and Hepatology, Zvezdara University Medical Center, 11000 Belgrade, Serbia; 3 Faculty of Medicine, University of Belgrade, 11000 Belgrade, Serbia

**Keywords:** safety, exposure-adjusted incidence rate, Kaplan–Meier curve, Crohn’s disease, adalimumab, monoclonal antibody

## Abstract

**Background/Objectives**: The safety profile of adalimumab in inflammatory bowel disease (IBD) has been established in clinical trials, but post-marketing surveillance remains important for detecting rare and late-onset adverse events (AEs). This study aimed to evaluate long-term safety and temporal dynamics of AEs in an IBD cohort treated with adalimumab, and to identify potential safety signals using the FDA Adverse Event Reporting System (FAERS). **Methods**: This retrospective single-center study included patients with IBD treated with adalimumab. Demographic, clinical, and treatment-related data, including AEs and newly identified extraintestinal manifestations (EIMs), were collected. Time-to-event (TTE) analyses were used to assess AEs temporal patterns and covariates associated with onset. FAERS disproportionality analysis was performed for cohort-identified AEs and EIMs. **Results**: A total of 217 patients with 591.29 patient-years (PYs) of exposure (up to 9.62 years) were included. AE incidence was 106.6/100 PY. Time to first AE was described using the Kaplan–Meier method, and additionally the Weibull distribution, with an estimated median time of 11.4 months (95% CI: 9.7–13.5). The most frequent AEs were infections (32.30/100 PY), hepatic events (9.98/100 PY), and cutaneous manifestations (8.46/100 PY), characterized by TTE analysis. Serious outcomes, including malignancies and demyelinating events, were rare. Several unlisted MedDRA preferred terms, including infectious, cutaneous, vascular, proliferative, and musculoskeletal events, emerged as FAERS signals. **Conclusions**: This study provides long-term real-world evidence supporting the established safety profile of adalimumab in IBD. First AEs occurred throughout treatment exposure with lower hazard over time. Isolated FAERS signals, including vascular occlusive events, may warrant further investigation and continued pharmacovigilance.

## 1. Introduction

Inflammatory bowel diseases (IBDs), comprising Crohn’s disease (CD) and ulcerative colitis (UC), represent chronic conditions characterized by immune-mediated inflammation of the gastrointestinal tract [1]. With global prevalence now exceeding 6.8 million cases and constantly increasing, IBD represents a growing burden on healthcare systems worldwide [2].

Tumor necrosis factor alpha (TNF-α) is a central mediator of inflammation in IBD pathogenesis [3], with elevated levels observed in the intestinal mucosa of affected patients [4]. The development of anti-TNF-α agents has changed the management and clinical course of IBD. Adalimumab, a fully human IgG1 monoclonal antibody that neutralizes TNF-α [5], received Food and Drug Administration (FDA) approval for the treatment of moderately to severely active CD in 2007, and for the treatment of moderately to severely active UC in 2012 [6,7]. The efficacy of adalimumab in inducing and maintaining remission has been demonstrated in multiple pivotal trials in both moderate-to-severe CD [8,9,10,11] and UC [12,13,14].

Comprehensive data from clinical trials, pooled safety data and meta-analyses consistently showed that adalimumab is well-tolerated in these patients. The incidence of adverse events (AEs), serious adverse events (SAEs), and AEs leading to discontinuation, were similar to or lower than placebo across induction and maintenance phases, with no new safety concerns identified during long-term treatment [6,8,9,10,11,12,13,14,15,16,17]. Infections represent the most common AEs associated with adalimumab, due to the immunosuppressive nature of TNF-α inhibitors [18]. However, the overall incidence of serious infections in adalimumab-treated patients has been stable and relatively low (approximately 6.7 events per 100 patient-years (PYs) in CD and 3.5 events per 100 PY in UC) [19], with meta-analyses confirming no significant difference in the rates of serious infections between placebo- and anti-TNF-α-treated patients [20,21]. Malignancies were also infrequent, with most malignancy rates comparable to the general population, although a slightly elevated risk of non-melanoma skin cancer has been observed, particularly in patients receiving concomitant immunosuppressive therapy [19,22].

Clinical trials often lack sufficient power to identify rare AEs and enroll a highly selective group of patients. Consequently, many individuals receiving biologic treatments for IBD in real-world settings are not represented in these clinical studies [23]. Post-approval safety of adalimumab has been researched in a prospective, observational 6-year PYRAMID registry involving over 5000 patients. As a result, no new safety findings were reported, and the observed incidence of lymphoma was lower than the estimated background rate [24]. These findings are consistent with results from multiple observational studies, which supported the safety profile established in clinical trials [25,26,27,28,29,30].

However, continued post-marketing surveillance is necessary, particularly for detecting rare AEs and those with longer latency periods. This is especially important in IBD, which requires long-term treatment and continuous monitoring for emerging safety signals. In addition to the identification of AEs, increasing attention has been directed toward assessing the temporal dynamics of their occurrence, including time-to-event (TTE), as well as exploring the potential influence of demographic, clinical, and treatment-related variables on these dynamics over time. Given the limited number of studies that systematically examine the impact of these factors on the temporal dynamics of the most common AEs in clinical practice, such analyses may contribute to a more detailed understanding of the adalimumab safety profile and generate hypotheses regarding patient characteristics potentially associated with AE risk.

A recent study highlighted the need to incorporate both active and passive pharmacovigilance methods to adequately assess the safety profile of anti-TNF agents [31]. Spontaneous reporting databases, such as the FDA Adverse Event Reporting System (FAERS), represent an important source of data for post-marketing safety monitoring [32]. Therefore, the aim of our study is to evaluate AEs associated with adalimumab in our patient cohort, to characterize TTE profiles and explore potential covariate effects, and to complement these findings by identifying potential safety signals using data from the FAERS.

## 2. Results

In this single-center study, a total of 2603 medical reports from 217 patients were analyzed. The majority had CD (89.86%), and 48.85% were male. The median age at the time of adalimumab initiation was 37 years (interquartile range (IQR): 27–45), with a median disease duration of 7 years (IQR: 2–13) prior to treatment initiation. The majority of patients had been previously exposed to immunosuppressive therapy (90.78%) and corticosteroids (69.12%), while 34.56% had prior exposure to anti-TNF-α therapy. The remaining baseline demographics and clinical characteristics of the patients, overall and by disease type (CD vs. UC) are presented in Table 1.

**Figure 1:**
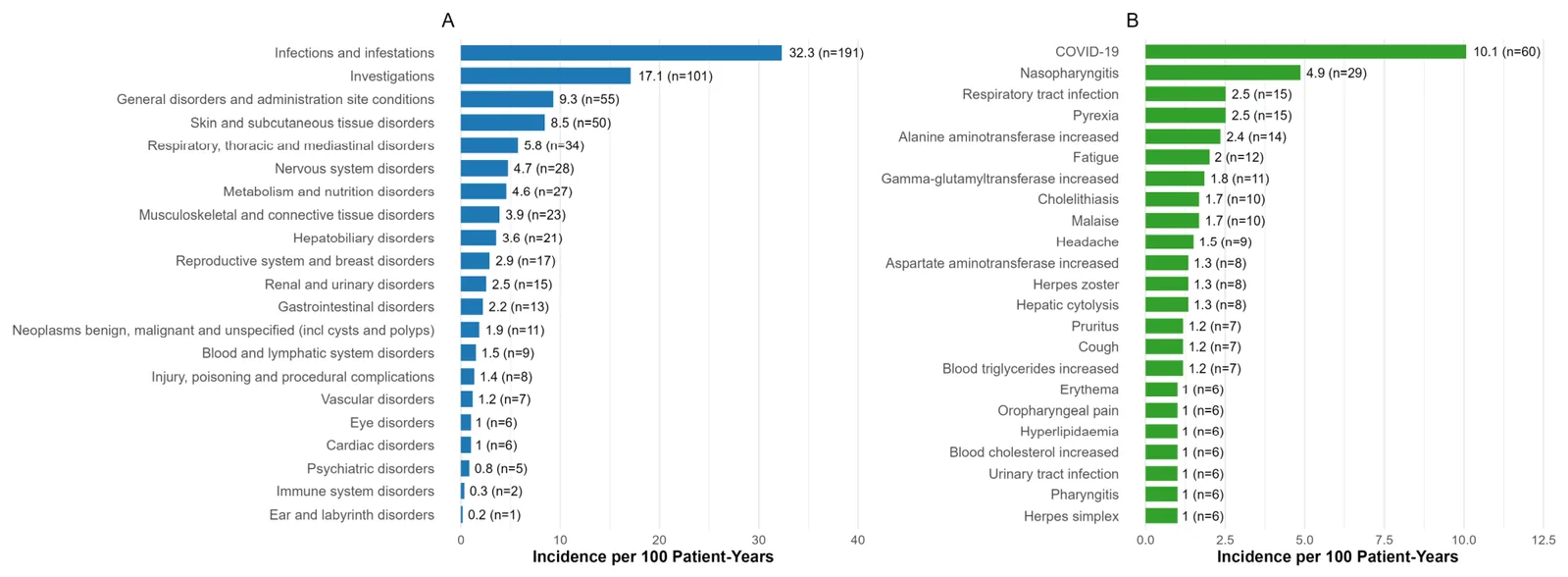
Incidence rates per 100 PY of reported events: (**A**) all events by Medical Dictionary for Regulatory Activities (MedDRA) system organ class (SOC); (**B**) most frequently reported events by MedDRA preferred term (PT).

The total adalimumab exposure in this study was 591.29 PY, with a median exposure of 888 days (IQR: 365–1457). A total of 630 events (106.55 events/100 PY) were recorded, with 168 out of 217 patients experiencing at least one AE. The most frequently reported events per Medical Dictionary for Regulatory Activities (MedDRA) system organ classes (SOCs) were infections and infestations (*n* = 191; 32.30 events/100 PY), investigations (*n* = 101; 17.08 events/100 PY), general disorders and administration site conditions (*n* = 55; 9.30 events/100 PY), skin and subcutaneous tissue disorders (*n* = 50; 8.46 events/100 PY), respiratory, thoracic and mediastinal disorders (*n* = 34; 5.75 events/100 PY) and nervous system disorders (*n* = 28; 4.74 events/100 PY). The most frequently reported events per MedDRA PT were COVID-19 (*n* = 60; 10.15 events/100 PY), nasopharyngitis (*n* = 29; 4.90 events/100 PY), respiratory tract infection (*n* = 15, 2.54 events/100 PY), pyrexia (*n* = 15, 2.54 events/100 PY), alanine aminotransferase increased (*n* = 14; 2.37 events/100 PY), fatigue (*n* = 12; 2.03 events/100 PY), and gamma-glutamyltransferase increased (*n* = 11; 1.86 events/100 PY). Incidence rates of all events by MedDRA SOC and of the most frequently reported events by PT are presented in Figure 1.

Out of the total number of events, 35 AEs occurred during the induction phase and 593 during the maintenance phase; two malignancies were detected after adalimumab discontinuation, in line with the study methodology. All AEs by MedDRA SOC, PT, and treatment phase, along with incidence rates per 100 PY, are presented in Supplementary Table S1.

Adalimumab was discontinued in 17 patients due to AEs, with 22 AEs reported as reasons for discontinuation. The most commonly involved SOCs were infections and infestations, general disorders and administration site conditions, musculoskeletal and connective tissue disorders, skin and subcutaneous tissue disorders, and nervous system disorders. Reported events leading to discontinuation included infections, injection-site reactions, lupus-like syndrome, dermatological reactions, demyelinating events, and ischaemic cerebral infarction. In single cases, treatment was also discontinued due to thrombocytopenia and brain neoplasm. Among the 17 patients who discontinued adalimumab due to AEs, 12 had previously discontinued at least one other IBD therapy because of AEs. Five had prior exposure to infliximab, including three patients in whom infliximab had also been discontinued due to AEs.

Causality assessment was not available for the majority of events. However, eight events in five patients were assessed by the treating physician as at least possibly related to adalimumab, including rash, paresthesia with demyelination, lupus-like syndrome, injection-site hypersensitivity, and dermatological reactions including dermatitis psoriasiform, pruritus, and alopecia.

**Figure 2:**
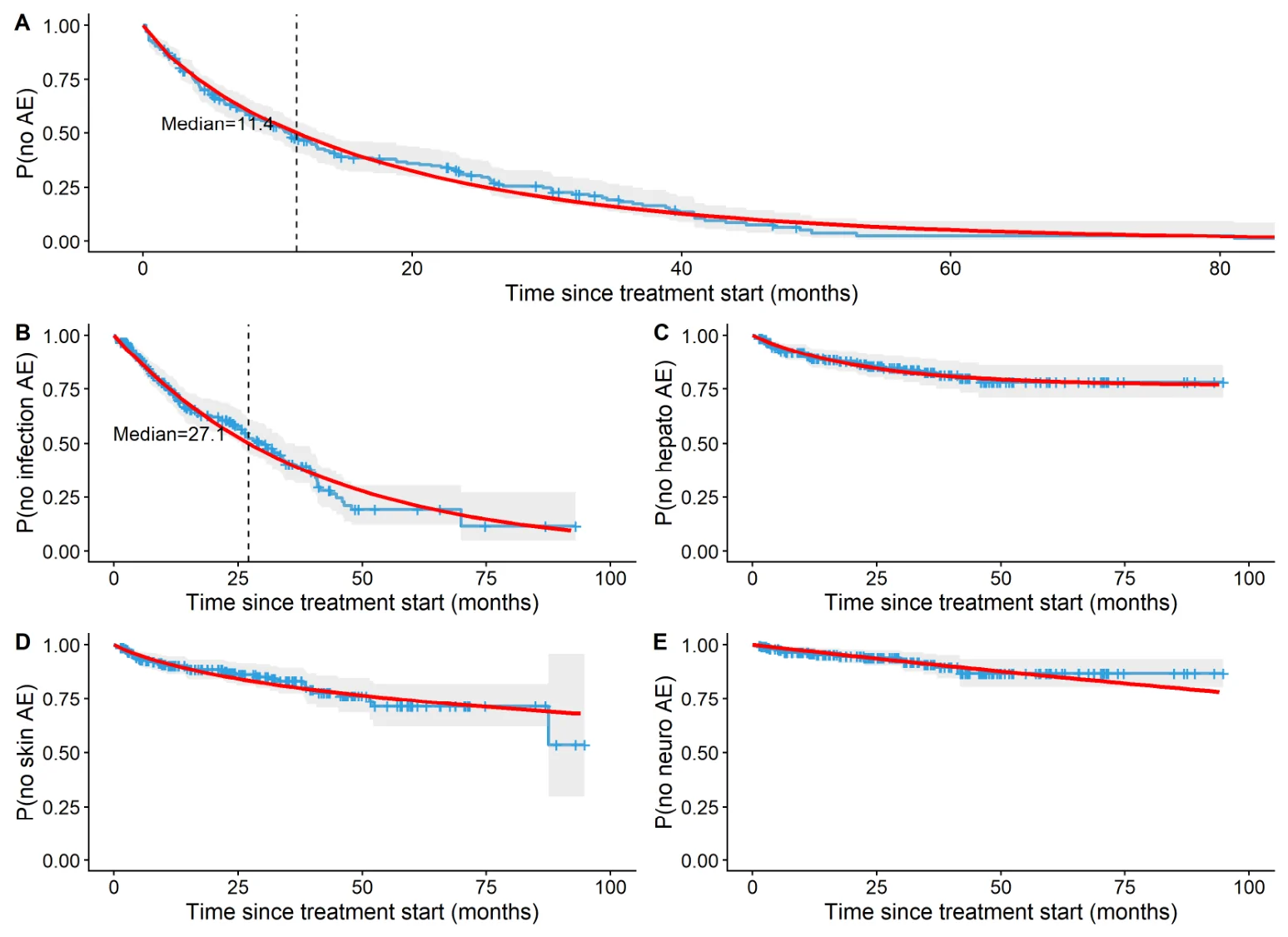
Kaplan–Meier and parametric time-to-event (TTE) models describing time to first adverse event (AE) and system organ class (SOC)-specific AEs: (**A**) overall first AEs; (**B**) infections and infestations; (**C**) hepatic-related events; (**D**) skin and subcutaneous tissue disorders; (**E**) nervous system disorders. Solid red lines represent model-fitted survival functions, while blue step functions represent Kaplan–Meier estimates. Gray areas indicate 95% confidence intervals. Median time-to-event estimates (dashed line) are reported where applicable.

Kaplan–Meier and parametric TTE analyses were performed to characterize the timing of first and category-specific AEs during follow-up. The study cohort consisted of 217 patients, each contributing a single treatment episode to the TTE analysis. As clinically relevant and relatively frequent SOCs, we identified infections and infestations, skin and subcutaneous tissue disorders, nervous system disorders, and hepatic-related AEs. Although hepatobiliary disorders alone showed a lower number of events, when combined with hepatic laboratory abnormalities classified under the investigations SOC (including increased alanine aminotransferase, aspartate aminotransferase, bilirubin, gamma-glutamyl transferase, and other hepatic enzyme abnormalities), the total number of hepatic-related events became clinically meaningful, with 59 events corresponding to an incidence of 9.98/100 PY. In contrast, the SOCs investigations and general disorders and administration site conditions were not selected for further TTE analysis due to their broad and heterogeneous nature. Respiratory, thoracic and mediastinal disorders were also excluded, as most events reflected nonspecific symptoms related to respiratory tract infections. Kaplan–Meier curves demonstrated differences in TTE profiles across AE categories, with corresponding survival functions shown in Figure 2.

Among the evaluated distributions, the Weibull model provided the best fit for time to first AE, as indicated by the lowest Akaike Information Criterion (AIC = 1304.4). The fitted model yielded a shape parameter of 0.86 (95% confidence interval (CI): 0.76–0.97), and a scale parameter of 17.49 months (95% CI: 14.66–20.85), as shown in Figure 2A. The estimated median time to first AE was 11.4 months (95% CI: 9.7–13.5 months). A total of 168 events were observed across 217 patients, while 49 intervals were censored during a total follow-up time of 3018.6 patient-months.

The TTE profile for infections and infestations was best described by an exponential model, with an estimated median TTE of 27.1 months (95% CI: 22.3–32.7 months). This model indicated an approximately constant hazard of infection over time (Figure 2B). For hepatic AEs, the Gompertz provided the best fit, suggesting a time-dependent hazard pattern with a decreasing risk of event occurrence over time (Figure 2C). Skin and subcutaneous tissue disorders were best characterized by a log-normal model (Figure 2D), although the Weibull and log-logistic models showed very similar fits. For nervous system disorders the exponential distribution provided the best fit (Figure 2E); however, only 17 events were observed during follow-up, indicating that these AEs were infrequent in the study population. This model indicated an approximately constant hazard of neurological AE occurrence over time. Most neurological and hepatic-related events occurred within approximately 40–44 weeks of follow-up.

To evaluate the potential impact of delayed AE ascertainment, the median interval between clinical contacts was calculated and was 1.61 months (IQR 0.92–3.68 months). Sensitivity analyses were subsequently performed by treating each AE as interval-censored within a one-month window preceding the documentation date, representing a conservative scenario in which the true onset could have occurred up to one month before recording. Across all AE categories, the best-fitting parametric distribution remained unchanged. For hepatic, skin, and neurological AEs, estimated cumulative incidence differed by no more than 0.7 percentage points at any evaluated landmark. For the two endpoints with directly estimable median TTE, the estimated median changed by 1.2 months for overall AEs (<10%) and by 0.3 months for infections (≈1%). Therefore, the TTE estimates were robust to the ascertainment scheme.

We evaluated multiple factors in Kaplan–Meier univariate analysis and identified several statistically significant differences between survival curves. However, some findings lacked clear clinical plausibility and were inconsistent with previously published data. Given the limited number of events within certain SOC categories, these results should be interpreted cautiously, as they may reflect the relatively small sample size, the absence of adjustment for multiple comparisons, and observational follow-up design.

**Figure 3:**
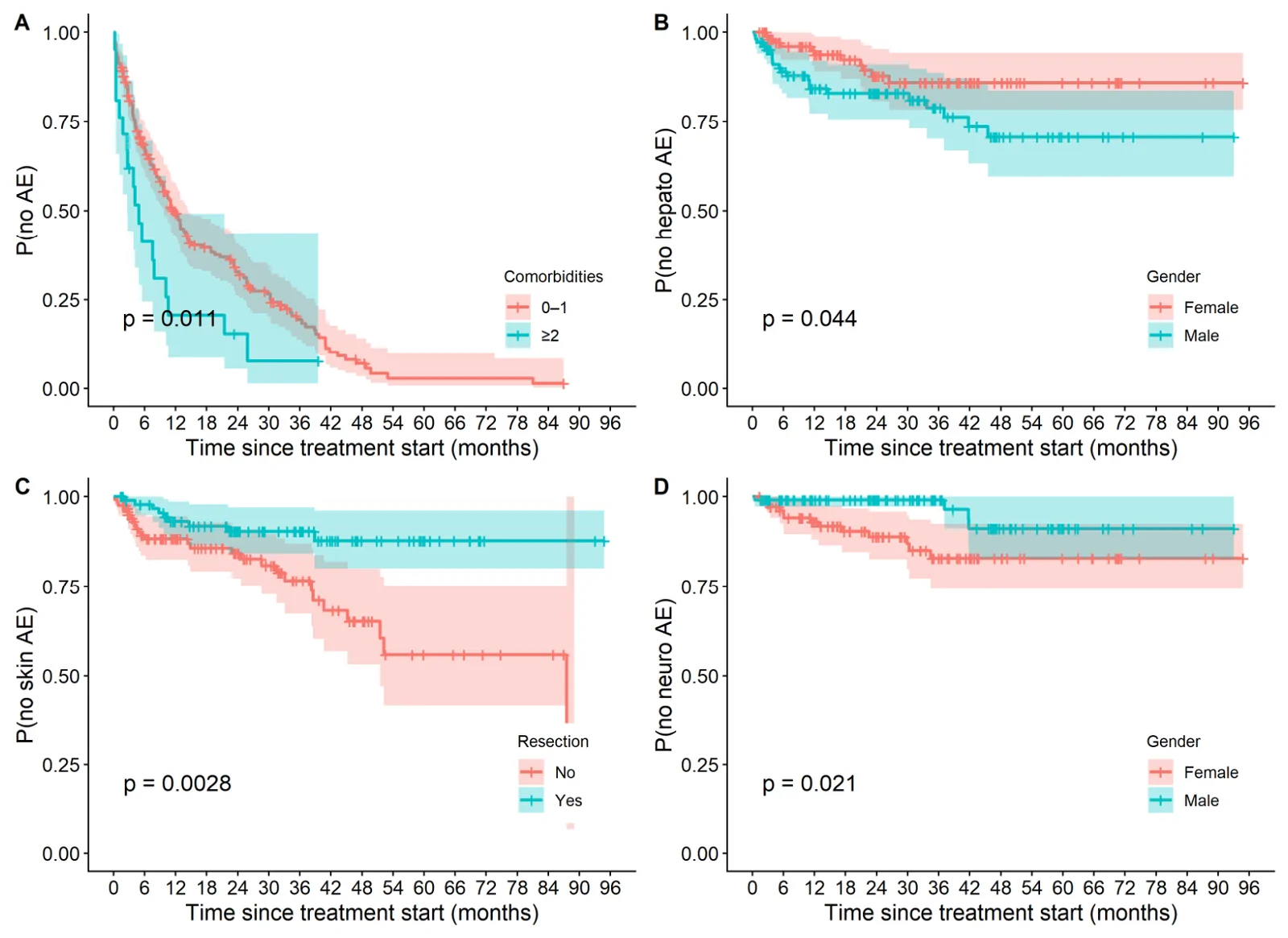
Kaplan–Meier survival curves for selected adverse events (AEs) stratified by significant covariates in log-rank tests. (**A**) Number of comorbidites on time to first AE; (**B**) gender on hepatic-related AEs; (**C**) resection on skin and subcutaneous tissue disorders; (**D**) gender on nervous system disorders. Step functions represent Kaplan–Meier estimates. Shaded areas indicate 95% confidence intervals.

Among the clinically more plausible findings, patients with ≥2 comorbidities had a significantly shorter time to first AE compared with those with ≤1 comorbidity in Kaplan–Meier analysis (log-rank *p* = 0.011), as shown in Figure 3A. In risk analysis, this association was also statistically significant, with higher hazard observed in patients with ≥2 comorbidities (HR: 1.85, 95% CI: 1.13–3.03; *p* = 0.015), suggesting association between comorbidity burden and AE risk.

Male gender was associated with a significantly shorter time to hepatic AEs compared with female gender in Kaplan–Meier analysis (log-rank *p* = 0.044, Figure 3B). In risk analysis, male gender showed a similar direction of effect, with a higher estimated hazard of hepatic AEs (HR 1.98, 95% CI: 0.95–4.11; *p* = 0.067), although this association did not reach statistical significance.

Previous intestinal resection was associated with a significantly longer time to skin-related AEs in Kaplan–Meier analysis (log-rank *p* = 0.0028, Figure 3C), and, a consistent protective association was observed in risk analysis, with lower hazard of skin-related AEs in patients with prior intestinal resection (HR: 0.34, 95% CI: 0.16–0.72; *p* = 0.005). In addition, female gender was associated with a significantly shorter time to neurological AEs compared with male gender in Kaplan–Meier analysis (log-rank *p* = 0.021, Figure 3D). In risk analysis, male gender was associated with a lower hazard of neurological AEs (HR: 0.32, 95% CI: 0.10–0.98; *p* = 0.046), indicating a consistent association across both approaches.

A total of 111 new EIMs were recorded, with an overall incidence of 18.77/100 PY. The most frequently reported new EIM was arthralgia (*n* = 51, 8.63/100 PY), followed by anemia-related events (*n* = 33, 5.58/100 PY), comprising anemia, iron deficiency anemia, and normocytic anemia. New EIMs detected in our cohort that met the criteria for signals of disproportionate reporting in the FAERS database included iridocyclitis, uveitis, arthralgia, joint stiffness, joint swelling, musculoskeletal stiffness, spinal pain, radiculopathy, erythema nodosum, psoriasis, and pyoderma gangrenosum; all assessed as unlisted according to adalimumab USPI, except psoriasis. Newly identified EIMs, along with the incidence and disproportionality analyses based on FAERS data, are presented in Supplementary Table S2.

**Figure 4:**
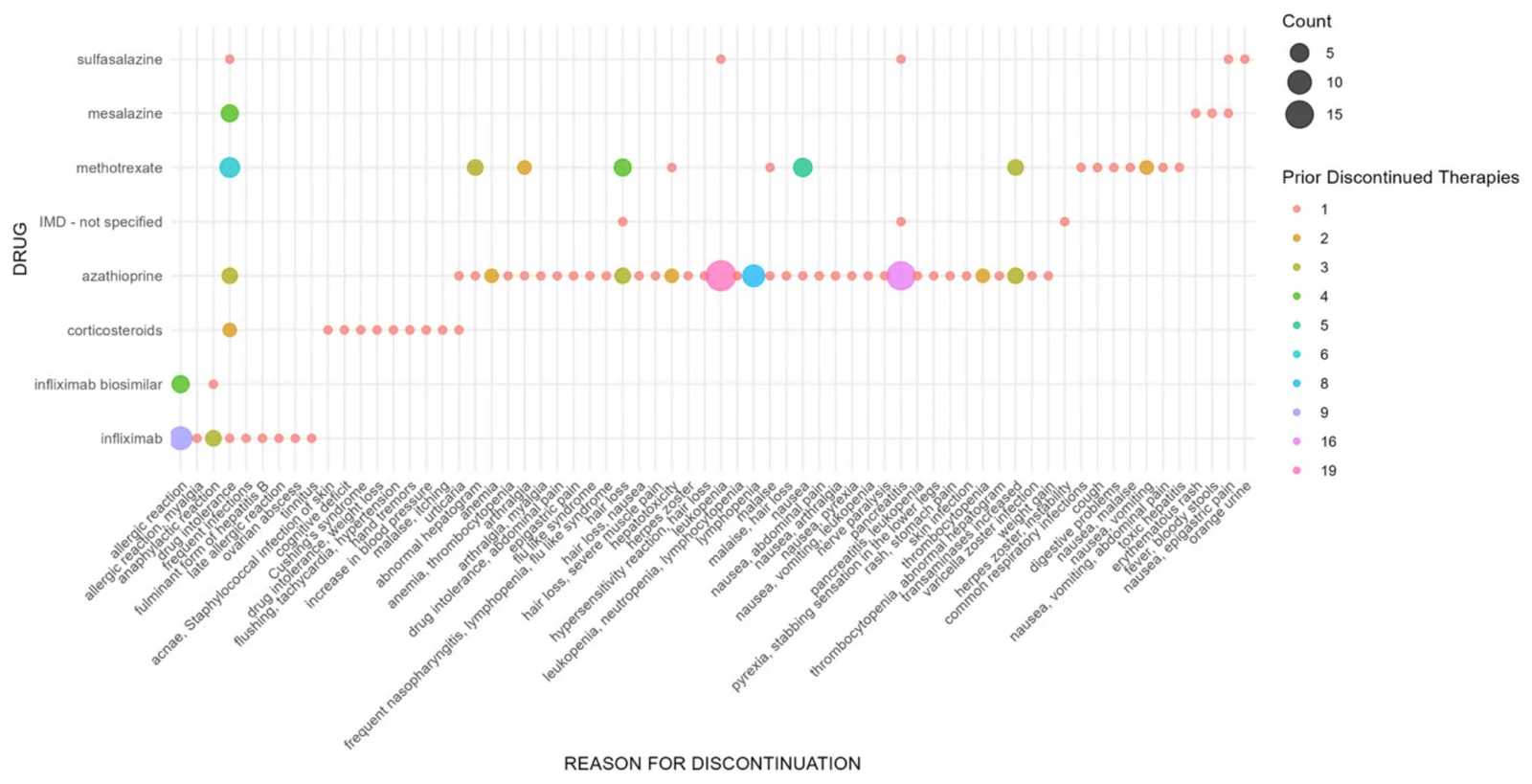
Treatments for inflammatory bowel disease (IBD) previously discontinued due to adverse events (AEs) in our patient cohort, along with reasons for discontinuations.

In our patient cohort, a total of 618 prior IBD therapies were recorded, with a median of three therapies per patient (IQR: 2–4). Of these, 359 were discontinued (median of two per patient, IQR: 1–2), of which 170 were discontinued due to AEs (median of one per patient, IQR: 0–1). The most commonly discontinued treatment due to AEs was azathioprine (*n* = 87), most frequently due to leukopenia (*n* = 22) and pancreatitis (*n* = 17), followed by methotrexate (*n* = 33), most commonly due to nausea (*n* = 8), and infliximab (*n* = 19), most commonly due to allergic reactions (*n* = 14), as shown in Figure 4.

**Figure 5:**
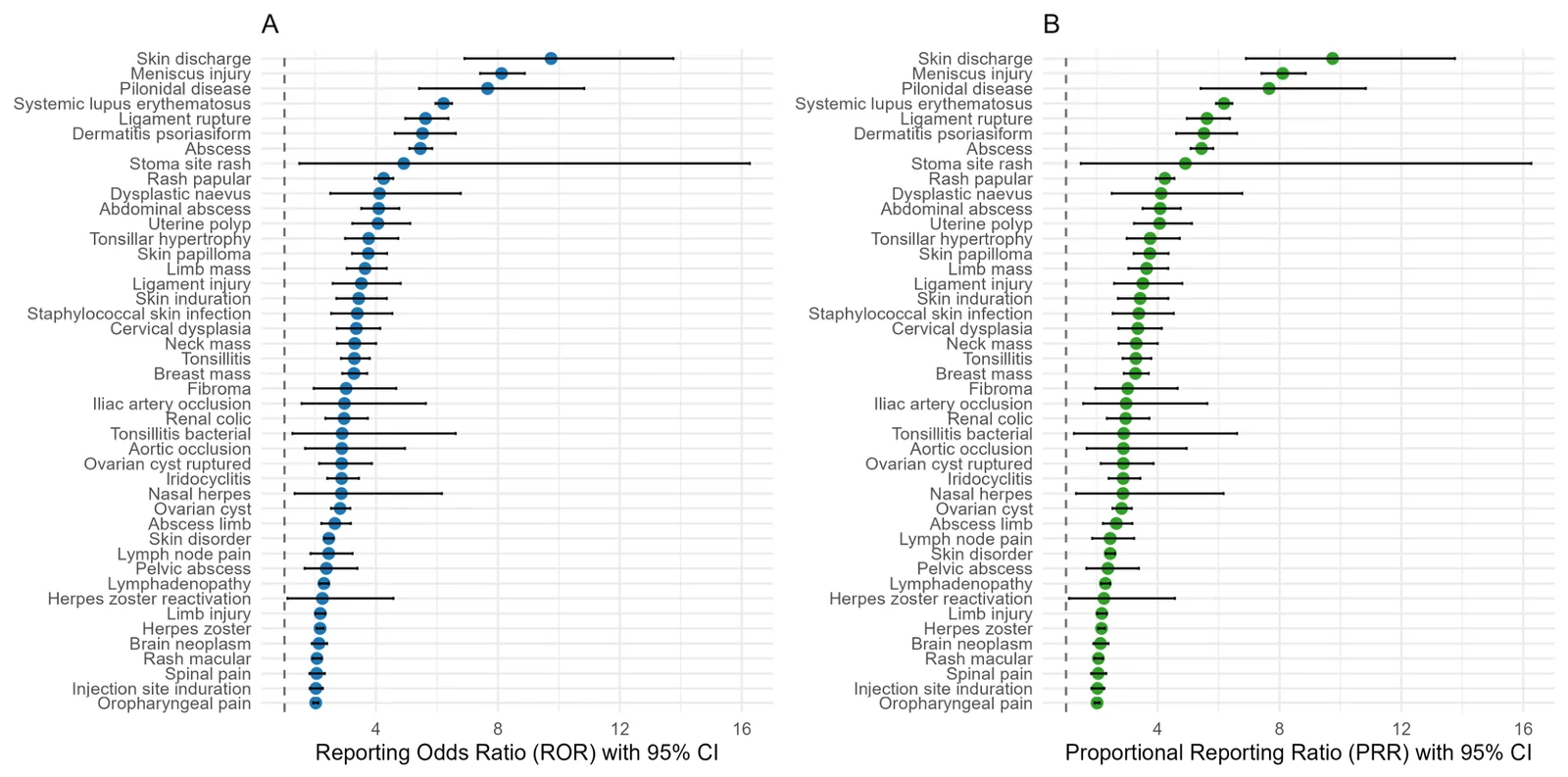
Disproportionality analysis results for unlisted Medical Dictionary for Regulatory Activities (MedDRA) preferred terms (PTs) meeting the criteria for signals of disproportionate reporting: (**A**) ROR with 95% CI; (**B**) PRR with 95% CI. CI: confidence interval; PRR: proportional reporting ratio; ROR: reporting odds ratio.

Results of the disproportionality analysis for all PTs reported in our cohort are presented in Supplementary Table S3, together with the listedness assessment according to the adalimumab USPI. All PTs that met the criteria for signals of disproportionate reporting and assessed as unlisted according to the USPI are presented in Figure 5.

## 3. Discussion

This study provides important insights into the long-term safety of adalimumab, addressing the limited availability of extended follow-up data in the literature [15,33], with patients followed for up to 9.62 years and 14.29% of patients having more than 5 years of treatment exposure.

Compared with pooled clinical trial data [15,33], both CD and UC patients in our cohort were of similar age (CD: 36 vs. 37.0 years; UC: 38 vs. 41.0 years). CD patients had a shorter time from diagnosis to adalimumab initiation (7 vs. 10.3 years), while the interval was similar in UC patients (8.5 vs. 8.0 years). This may reflect the more limited role of adalimumab in UC compared with CD in real-world clinical practice. Differences in treatment patterns were also observed, including lower concomitant corticosteroid exposure in CD (36.9% vs. 48.4%) and markedly lower concomitant immunosuppressant use in UC (36.4% vs. 71.4%), which may be related to differences in treatment strategies between clinical practice and trial settings. Compared with the PYRAMID registry (CD only) [24], patients in our cohort were of similar age (36 vs. 37.8 years), with the most notable difference being lower prior biologic exposure (30.3% vs. 56.8%).

The overall incidence of AEs in our cohort (106.6/100 PY; 77.4% of patients) was markedly lower than that reported in pooled clinical trial data (605/100 PY for CD and 361/100 PY for UC) [19], but higher than in the PYRAMID registry (36.7/100 PY; 47.6% of patients) [24]. The lower AE rates compared with clinical trials likely reflect the retrospective design of our study and potential underreporting, as AEs were captured from discharge summaries and may not have been systematically recorded, particularly when occurring outside hospitalization. In contrast, the higher AE incidence compared with the PYRAMID registry may be related to differences in patient populations and methods of AE collection between the two cohorts. While the PYRAMID registry relied on routine registry-based pharmacovigilance reporting, our study retrospectively captured potential AEs from all routinely documented clinical symptoms and findings recorded during regular follow-up visits in tertiary care IBD units, which may have resulted in a greater number of recorded events. Beyond incidence estimates, the TTE analysis provided additional insight into the temporal pattern of AE occurrence. The Weibull model showed the best fit for the observed data and estimated a median time to first AE of 11.4 months, indicating that half of the patients remained free of AEs beyond approximately one year of follow-up. The estimated shape parameter suggested high early risk of experiencing a first AE, which gradually declines over time. Kaplan–Meier analysis identified a significantly shorter time to first AE among patients with a higher comorbidity burden, and this association was also confirmed in the risk analysis. This finding should be assessed with caution due to the limited number of events in the study cohort and the absence of adjustment for multiple comparisons.

The most frequently reported events by MedDRA SOC were infections and infestations, while the most common MedDRA PTs were mild respiratory infections, including COVID-19, nasopharyngitis, and respiratory tract infection, consistent with findings from clinical trials and the PYRAMID registry [15,24]. The incidence of these events in our cohort was lower than that reported in clinical trials (32.30/100 PY vs. 152/100 PY) [34], similarly to the overall AE incidence described above. The relatively frequent occurrence of COVID-19-related events in our cohort should also be interpreted in the context of the study period overlapping with the COVID-19 pandemic. Other infectious AEs at the MedDRA PT level with an incidence higher than 1/100 PY, including herpes zoster, herpes simplex, urinary tract infection, and pharyngitis, had also been previously reported in clinical trials and therefore did not represent unexpected findings in our cohort [18]. Besides herpes virus-related infections, no other opportunistic infections were identified in our patient cohort. Beyond incidence-based observations, the TTE analysis suggested a relatively constant hazard of infection-related events over follow-up, consistent with an exponential model and indicating a stable risk of infections throughout the treatment period rather than a clearly defined period of increased susceptibility (Figure 2B).

The second most frequently reported group of events were hepatic-related events, including alanine aminotransferase increased, gamma-glutamyltransferase increased, aspartate aminotransferase increased, cholelithiasis, and hepatic cytolysis. Such findings are consistent with clinical trial data, where alanine aminotransferase elevations ≥3× upper limit of normal were observed in a small proportion of patients [35], and with prospective observational IBD data showing that most transaminase elevations are transient and rarely clinically significant [36]. Rare cases of clinically relevant drug-induced liver injury have also been described in the context of anti-TNF-α therapy [37], supporting continued monitoring of hepatic parameters during treatment. The temporal pattern of hepatic events appeared relatively distributed over time, with accumulation throughout approximately 44 months of follow-up and without a distinct early peak. The Gompertz model suggested a decreasing hazard of hepatic events over time, indicating that the risk was higher earlier during follow-up and gradually declined over time. Because hepatic adverse events were ascertained at clinical contacts, recorded onset may postdate true onset for events detected on routine laboratory monitoring; a sensitivity analysis treating events as interval-censored between the last contact and the documentation date changed the estimated cumulative incidence by no more than 0.3 percentage points at 12, 24, 36 and 48 months, confirming that the reported estimates are robust to the timing of ascertainment. Gender-related differences observed for hepatic events were not clearly aligned with previously published data and should be interpreted with caution (Figure 3B). Overlapping confidence intervals suggest substantial uncertainty, likely due to the small number of events and the absence of adjustment for multiple comparisons, making this a hypothesis-generating finding.

Skin and subcutaneous tissue disorders were among the most frequently reported AEs in our cohort, with pruritus and various rash-related manifestations being predominant, together with psoriasis, which was classified as an EIM. Similar findings have been reported in observational studies of adalimumab-treated IBD patients, where skin disorders represented one of the most commonly reported AE categories [27]. In addition, anti-TNF-α therapy has been associated with paradoxical inflammatory skin reactions, most commonly psoriasiform and eczematous lesions [34]. Registry data, including the PYRAMID study, further support a low but clinically relevant incidence of psoriasis-related events during adalimumab treatment, occasionally leading to treatment discontinuation [24]. TTE analysis indicated that skin and subcutaneous tissue disorders were best described by a log-normal model, although the Weibull model showed a very similar fit, suggesting that the risk of skin-related AEs was not constant over the course of follow-up. Previous intestinal resection was associated with a lower cumulative incidence of skin-related AEs, as indicated by both Kaplan–Meier and risk analyses, although the mechanism underlying this association remains unclear and may potentially involve differences in disease phenotype or gut–skin immune interactions following surgery. Given the limited number of events and the absence of adjustment for multiple comparisons, this finding should be interpreted cautiously.

Neurological AEs in our cohort were predominantly represented by headaches, while clinically more notable events included paresthesia and one case of demyelination. Similar neurological complications, including demyelinating disorders and peripheral neuropathies, have previously been described during anti-TNF-α therapy, although they are considered rare AEs [6,38]. Neurological AEs were infrequent in the study population, limiting the strength of conclusions regarding their temporal pattern. Female gender was associated with earlier occurrence of neurological AEs; however, given the small number of events and the absence of adjustment for multiple comparisons, this association should be considered exploratory and interpreted cautiously (Figure 3D). Although this finding is not clearly supported by existing anti-TNF safety data, the higher background prevalence of demyelinating disorders and related neurological manifestations among women may represent one possible explanation.

Among other frequently reported AEs outside the selected SOC categories, pyrexia, fatigue, malaise, cough, oropharyngeal pain, and lipid metabolism abnormalities, including hyperlipidemia and increased cholesterol and triglyceride levels, were also observed. These findings were generally nonspecific, consistent with the known safety profile of adalimumab, and have previously been reported in both clinical trials and real-world studies of anti-TNF-α therapy.

The most frequently observed EIMs in our cohort were arthralgia- and anemia-related events, which are consistent with those most commonly reported in the literature [34,39]. Several unlisted EIMs identified in our cohort, including iridocyclitis, uveitis, arthralgia, erythema nodosum, and pyoderma gangrenosum, also appeared as disproportionality signals in FAERS; however, these are well-recognized EIMs of underlying IBD and therefore are unlikely to represent adalimumab-specific effects. Joint, spine, and musculoskeletal symptom-related signals may also be influenced by underlying rheumatologic conditions for which adalimumab is prescribed, particularly rheumatoid arthritis and axial spondyloarthritis.

No deaths were recorded in our cohort during the observation period. Three malignancies were identified, including pancreatic adenocarcinoma, hepatic cancer, and ovarian cancer. Malignancies have previously been reported in both clinical trials and observational studies of anti-TNF-α therapy; however, most available data have not demonstrated a significant increase in overall malignancy risk compared with background IBD populations [15,24]. Given the small number and heterogeneous nature of malignancies observed in our cohort, no conclusions regarding treatment-related risk can be drawn.

Discontinuations of adalimumab due to AEs were most commonly associated with infections, injection-site reactions, dermatological reactions, lupus-like syndrome, and neurological events, consistent with previously reported safety concerns during anti-TNF-α therapy. Most patients who discontinued adalimumab due to AEs had also previously discontinued at least one other IBD therapy because of AEs, while a subset had prior intolerance to infliximab. Patterns of discontinuation of previous IBD therapies were also consistent with their established safety profiles, particularly azathioprine-associated leukopenia and pancreatitis, methotrexate-associated nausea, and hypersensitivity reactions during infliximab therapy.

The unlisted MedDRA PTs identified as signals for adalimumab were grouped into clinically meaningful categories. Infectious signals included abscess and soft tissue infection-related events (abscess, abdominal abscess, pelvic abscess, abscess limb, pilonidal disease, and Staphylococcal skin infection), upper respiratory tract infections (tonsillitis and bacterial tonsillitis), herpes virus-related events (herpes zoster, herpes zoster reactivation, and nasal herpes), and nonspecific symptoms commonly associated with upper respiratory tract infections (oropharyngeal pain). Although not specifically listed for adalimumab, these findings are consistent with increased susceptibility to infections and viral reactivation associated with TNF-α blockade. Cutaneous and local inflammatory reactions included injection-site induration, stoma site rash, rash macular, rash papular, skin induration, skin discharge, skin disorder, and dermatitis psoriasiform. These likely reflect local inflammatory responses and paradoxical cutaneous reactions previously described with anti-TNF-α therapy. Musculoskeletal injury-related signals included ligament injury, ligament rupture, limb injury, and meniscus injury. These may reflect mechanical injury in the general population but may also be influenced by the underlying rheumatologic and musculoskeletal conditions for which TNF-α inhibitors are frequently prescribed, as well as changes in physical function after treatment initiation. These findings should be interpreted with caution, and further pharmacovigilance is warranted to clarify their potential relationship with therapy. Lymphoid and proliferative signals included lymphadenopathy, lymph node pain, neck mass, limb mass, and breast mass, as well as fibroma, skin papilloma, dysplastic naevus, cervical dysplasia, ovarian cyst, ovarian cyst ruptured, and uterine polyp). These represent a heterogeneous group of reactive lymphoid changes, benign proliferative lesions, cystic findings, and premalignant conditions. Given the role of TNF-α in immune regulation, chronic inflammation, and cellular proliferation, these signals may reflect altered inflammatory and proliferative pathways during TNF-α inhibitor therapy; however, their clinical relevance remains uncertain in the absence of detailed clinical data. Tonsillar hypertrophy likely reflects reactive lymphoid enlargement. Brain neoplasm was identified as an isolated clinically notable signal. Although lymphomas and non-melanoma skin cancers have been previously associated with TNF-α inhibitors, central nervous system neoplasms have not been specifically reported in this context. Vascular occlusive signals included aortic occlusion and iliac artery occlusion. These are likely multifactorial and influenced by underlying cardiovascular risk in chronic inflammatory disease, although rare thrombotic events have been reported with TNF-α inhibitors [40,41]. While TNF-α inhibitors may improve inflammatory disease control and potentially reduce cardiovascular risk, these findings highlight the need for continued monitoring and further investigation of rare vascular complications in this population. Additional isolated signals, renal colic and systemic lupus erythematosus (SLE), were identified. Renal colic likely reflects nephrolithiasis, which is a recognized AE for adalimumab. Although SLE itself is not listed for adalimumab, lupus-like syndrome is a listed adverse reaction associated with TNF-α inhibitor therapy, and numerous cases of TNF-α inhibitor-induced lupus have been described in the literature [42,43].

While most findings were consistent with the known safety profile and immunomodulatory effects of TNF-α inhibition therapy, several isolated signals, including vascular occlusive events, brain neoplasm, and SLE, may warrant further clinical consideration and continued pharmacovigilance.

This study has several limitations. It is a single-center retrospective analysis based on medical records, which may have led to incomplete documentation of AEs. The predominance of patients with CD and the relatively small number of patients with UC reflect the composition of our adalimumab-treated cohort and are consistent with several published real-world adalimumab cohorts [44,45]. Nevertheless, this imbalance limited the generalizability of safety findings to the UC population. Baseline differences between these two groups, including previous biologic exposure, should also be considered when interpreting the observed results. Therefore, while the presented findings provide insight into the safety profile of adalimumab in a real-world IBD population, the caution is warranted when extending these results to patients with UC. Additionally, exact onset dates were not available for all events, limiting the precision of TTE analyses, and seriousness and causality were not systematically assessed for most events. It should also be acknowledged that, as AEs were captured from discharge summaries and causality assessment was unavailable for the majority of events, the reported AEs should be interpreted primarily as temporally associated documented clinical events rather than confirmed adverse drug reactions. The relatively small number of events also limited more detailed subgroup and TTE analyses. Although concomitant therapies, including corticosteroids and immunomodulators, were evaluated as treatment-related covariates in the TTE analyses, their potential confounding effect cannot be excluded, particularly for infectious events, given the observational design and limited number of events. FAERS data are further limited by underreporting, lack of clinical detail, and absence of denominator data, which does not allow incidence estimation and limits interpretation of detected signals. The FAERS analysis was focused on AEs identified in the study cohort. Consequently, concordance between the cohort and FAERS findings should be interpreted with caution, as it represents complementary rather than independent evidence for the observed associations. Despite these limitations, the study provides long-term follow-up of adalimumab in a real-world tertiary IBD cohort with substantial treatment exposure. A strength of the study is the use of TTE analysis to describe temporal patterns of AEs, together with the combination of cohort data and FAERS signals, providing complementary perspectives on adalimumab safety.

## 4. Materials and Methods

A retrospective observational study was conducted based on the safety data of adalimumab in patients with IBD treated at Zvezdara University Medical Center in Belgrade, Republic of Serbia, between May 2014 and September 2024. The study protocol was approved by the Ethics Committee of the Zvezdara University Medical Center (approval number IRB00009457, dated 7 October 2022).

All adult patients (≥18 years) who received adalimumab therapy were included (*n* = 217), while pregnant and breastfeeding women were excluded. Patients were followed from treatment initiation until treatment discontinuation or the date of the last available clinical report if therapy had not been discontinued. Malignancies were recorded regardless of whether they occurred during the defined follow-up period.

The following patient characteristics were extracted from available medical records: demographic data (age at treatment initiation, gender, age at the time of diagnosis), clinical characteristics (underlying diagnosis (UC or CD), presence of EIMs at baseline, number of comorbidities, history and extent of surgical resection, Mayo score if available, presence of stoma, presence of perianal disease, and information on clinical, laboratory, and endoscopic remission), as well as treatment-related data (dose, drug concentration, treatment phase (induction or maintenance), information on whether the drug was originator or biosimilar, prior therapies including immunosuppressants, corticosteroids, and TNF-α inhibitors, previously discontinued therapies and reasons for discontinuation including AEs, as well as concomitant therapies).

An AE was defined as any unfavorable medical occurrence regardless of causal relationship with the drug [46]. In this study, all AEs were recorded from the available hospital reports, while symptoms related to worsening of the underlying disease were not considered AEs. Newly occurring EIMs after treatment initiation were also recorded. Drug exposure time was defined as the period from the first dose to treatment discontinuation or the last available medical record if the treatment was not discontinued. The incidence of AEs and new EIMs was expressed as the number of events per 100 PY of exposure. All AEs and new EIMs were coded using the Medical Dictionary for Regulatory Activities (MedDRA). The MedDRA (version 28.1; International Council for Harmonisation (ICH), Geneva, Switzerland), released in September 2025, was used for coding and analysis in this study. Each AE was further assessed to determine whether it was listed as an expected event in the official U.S. Prescribing Information (USPI) [35].

To evaluate whether AEs and new EIMs identified in this real-world cohort represented potential safety signals, a disproportionality analysis was performed using data from the FAERS with the utilization of OpenVigil 2.1, an open-access tool for pharmacovigilance data analysis [47]. Reports in which adalimumab was classified as the primary suspect drug were included, covering the period from 1 January 2007 to 31 December 2025. The full FAERS database for the corresponding period was used as the reference background. Reports were not restricted according to the indication for adalimumab treatment because indication data in FAERS are incompletely reported and inconsistently coded.

Disproportionality was assessed using the frequentist methods, the proportional reporting ratio (PRR) and the reporting odds ratio (ROR), based on 2 × 2 contingency tables comparing the observed number of drug–AE combinations of interest with all other drugs and events in the database [33]. The following formulas were used to calculate PRR and ROR for all events at MedDRA PT level:

(1) $$PRR=\frac{a\cdot (c+d)}{c\cdot (a+b)}$$

(2) $$ROR=\frac{a\cdot d}{b\cdot c}$$

where *a* denotes number of reports for the drug of interest and the AE of interest, *b* the number of reports for other AEs attributed to the drug of interest, *c* the number of reports for the AEs of interest for all other drugs, and *d* the number of reports for all other combinations of drugs and events in the database [33].

For the purpose of this study, an AE was considered a signal if predefined thresholds were met for both PRR (PRR ≥ 2, Chi-square ≥ 4, and number of reports ≥ 3) and ROR (lower bound of the 95% confidence interval > 1 and number of reports ≥ 3).

Previous therapies for IBD were also analyzed, including the average number of prior therapies, the number of discontinued therapies, and the number of therapies stopped due to AEs, while AEs leading to discontinuation were presented in more detail.

Descriptive statistics were used to summarize demographic and clinical characteristics, AEs, EIMs, and treatment history. Continuous variables were presented as mean ± standard deviation or median with IQR, depending on data distribution, which was assessed using the Shapiro–Wilk test. Categorical variables were presented as counts and percentages. Analyses were conducted using Microsoft Excel (version 2108; Microsoft Corporation, Redmond, WA, USA) and R (version 4.5.1; R Foundation for Statistical Computing, Vienna, Austria) in RStudio (version 2025.09.1+401; Posit Software, PBC, Boston, MA, USA), with appropriate packages.

TTE analyses were performed to evaluate the temporal occurrence of AEs, focusing on the first AE or groups of events with sufficient frequency for reliable estimation. For most AEs, exact onset dates were not available; therefore, the date of the medical record in which the AE was first documented was considered as the onset date. Patients were regularly monitored during scheduled follow-up visits and were instructed to promptly contact their gastroenterologist in the event of new symptoms or signs. Consequently, the recorded dates were considered a reasonable approximation of AE onset. Patients who did not experience AEs during follow-up were treated as right-censored, with the last available date used as the censoring time. All TTE analyses were repeated treating each event as interval-censored within a one-month interval preceding the documentation date, using nonparametric (Turnbull) and parametric interval-censored models. For the TTE analyses, only the first adalimumab treatment episode for each patient was included to ensure that each patient contributed a single observation.

Nonparametric TTE analysis was initially performed using the Kaplan–Meier method to estimate the probability of remaining event-free over time and to describe TTE profiles for overall AEs and selected AE categories. Univariate analyses of categorical covariates were conducted using Kaplan–Meier curves and compared using the log-rank test, while continuous variables were analyzed using the Cox proportional hazards model. Covariates evaluated in univariate TTE analyses included demographic characteristics (e.g., age, gender), clinical characteristics (e.g., primary diagnosis, disease duration, presence of stoma, presence of perianal disease, number of comorbidities), and treatment-related factors (e.g., prior therapies, concomitant use of immunosuppressants and/or corticosteroids). Statistical significance was defined as *p* < 0.05. No adjustment for multiple comparisons was applied, as these analyses were considered exploratory and intended to identify potential covariates associated with AE occurrence.

In addition, parametric TTE models were fitted to the observed event-time data to characterize the underlying distribution of AE occurrence. Exponential, Weibull, log-normal, log-logistic, and Gompertz distributions were evaluated for each AE category. Model selection was based on clinical plausibility, goodness-of-fit criteria including AIC and Bayesian information criterion (BIC) [48,49,50]. Survival probabilities were derived from the final selected models.

A stepwise covariate modeling strategy was pre-specified; however, only covariates demonstrating statistical significance in univariate screening were considered for inclusion in multivariable parametric TTE models. Due to the rather small sample size, limited number of events within several AE categories, and limited number of statistically significant covariates in the univariate analyses, covariate effects using parametric TTE modeling was not further pursued. The analyses and graphical outputs were performed using R (version 4.5.1; R Foundation for Statistical Computing, Vienna, Austria) with relevant packages (including survival, survminer, flexsurv, ggplot2).

For each AE category, a risk analysis was performed by constructing patient-level exposure groups and estimating event occurrence using a 2 × 2 contingency framework. TTE data were additionally analyzed using a Cox proportional hazards model to estimate HRs with corresponding 95% CIs for each covariate. Results are reported as HRs with 95% CIs and *p*-values to quantify the relative risk of AE occurrence between exposed and unexposed groups.

## 5. Conclusions

This study provides long-term real-world evidence on the safety of adalimumab in patients with IBD, with follow-up extending up to 9.62 years. AEs were most frequently related to infections, hepatic laboratory abnormalities, and cutaneous manifestations, while serious outcomes such as malignancy and neurological events were rare. No unexpected safety signals were identified in the cohort analysis. TTE analysis suggested that AEs may occur throughout treatment rather than predominantly during early exposure periods. Several isolated signals identified through FAERS analysis, including vascular occlusive events, brain neoplasm, and SLE, may warrant further investigation and continued pharmacovigilance.

## Figures and Tables

**Table 1:** Baseline demographic and clinical characteristics of patients overall and by disease type (CD vs. UC).

Variable	Overall (*n* = 217)	CD (*n* = 195, 89.89%)	UC (*n* = 22, 10.14%)
Age at time of adalimumab initiation, median (IQR)	37 (27–45)	36 (27–45)	38 (30.5–43.3)
≥65 years of age, *n* (%)	2 (0.92)	1 (0.51)	1 (4.55)
Male gender, *n* (%)	106 (48.85)	95 (48.72)	11 (50)
Resection, *n* (%)	95 (43.78)	95 (48.72)	0 (0)
Stoma, *n* (%)	16 (7.37)	16 (8.21)	0 (0)
Perianal disease, *n* (%)	39 (17.97)	38 (19.49)	1 (4.55)
EIM present at baseline, *n* (%)	72 (33.18)	59 (30.26)	13 (59.09)
Age at time of diagnosis, median (IQR)	27 (21–36)	27 (21–36.5)	29.5 (19.5–34.5)
Pediatric onset, *n* (%)	21 (9.68)	19 (9.74)	2 (9.09)
Number of years from diagnosis to adalimumab initiation, median (IQR)	7 (2–13)	7 (2–13)	8.5 (3.5–15)
Previous immunosuppressive therapy, *n* (%)	197 (90.78)	175 (89.74)	22 (100)
Previous exposure to anti-TNF-α agents, *n* (%)	75 (34.56)	59 (30.26)	16 (72.73)
Previous corticosteroids therapy, *n* (%)	150 (69.12)	128 (65.64)	22 (100)
Concomitant corticosteroids, *n* (%)	86 (39.63)	72 (36.92)	14 (63.64)
Concomitant immunosuppressive therapy, *n* (%)	131 (60.37)	123 (63.08)	8 (36.36)
Concomitant corticosteroids and immunosuppressive therapy, *n* (%)	46 (21.20)	41 (21.03)	5 (22.73)
Number of comorbidities per patient, median (IQR)	0 (0–1)	0 (0–1)	0 (0–1)
Total exposure (PY)	591.29	556.72	34.57
Exposure, years, median (IQR)	2.43 (1.00–3.99)	2.57 (1.15–4.07)	1.05 (0.63–2.32)
Maximum exposure	9.62	9.62	5.24
>2 years of exposure, *n* (%)	124 (57.14)	118 (60.51)	6 (27.27)
>5 years of exposure, *n* (%)	31 (14.29)	30 (15.38)	1 (4.55)

CD: Crohn’s disease; EIM: extraintestinal manifestation; IQR: interquartile range; *n*: number; PY: patient-years; TNF: tumor necrosis factor; UC: ulcerative colitis.

## Data Availability

Clinical data that support the findings of this study are not openly accessible due to ethical and privacy restrictions. FDA Adverse Event Reporting System (FAERS) database and source are freely available: OpenFDA is freely accessible at https://api.fda.gov/drug/event.json (accessed on 14 February 2026). OpenVigil FDA can be used or downloaded at http://openvigil.sourceforge.net (accessed on 14 February 2026).

## Supplementary Materials

The following supporting information can be downloaded at https://www.mdpi.com/article/10.3390/ph19081239/s1, Table S1: All adverse events by MedDRA SOC, PT, and treatment phase, with incidence rates per 100 PYs. Table S2: New EIMs by MedDRA SOC and PT, with incidence, disproportionality analysis of FAERS data, and listedness assessment. Table S3: Disproportionality analysis for selected adverse events and their listedness according to the adalimumab USPI.

## Abbreviations

The following abbreviations are used in this manuscript:

AE	Adverse Event
AIC	Akaike Information Criterion
BIC	Bayesian Information Criterion
CD	Crohn’s Disease
EIM	Extraintestinal Manifestation
FAERS	FDA Adverse Event Reporting System
FDA	Food and Drug Administration
HR	Hazard Ratio
IBD	Inflammatory Bowel Disease
IQR	Interquartile Range
MedDRA	Medical Dictionary for Regulatory Activities
PRR	Proportional Reporting Ratio
PT	Preferred Term
PY	Patient Years
ROR	Reporting Odds Ratio
SAE	Serious Adverse Event
SLE	Systemic Lupus Erythematosus
SOC	System Organ Class
TNF-α	Tumor Necrosis Factor Alpha
TTE	Time-To-Event
UC	Ulcerative Colitis
