# Supplementary material for: Adalimumab Safety in Inflammatory Bowel Disease: A Clinical Cohort Study with Time-to-Event Analysis and FAERS Signal Assessment
Source: Pharmaceuticals (Basel). 2026 Aug 6;19(8):1239. doi: 10.3390/ph19081239 (PMC13516148; doi:10.3390/ph19081239)
Supplement: Supplementary file 1 [file pharmaceuticals-19-01239-s001.zip › pharmaceuticals-4419205-supplementary.pdf]

Adalimumab Safety in Inflammatory Bowel Disease: A Clinical Cohort Study with Time-to-Event Analysis and FAERS Signal Assessment

Bojana Milašinović [1], Srđan Marković [2,3], Sandra Vezmar Kovačević [1], Petar Svorcan [2,3], Branislava Miljković [1] and Katarina Vučićević [1,*]

1 Department of Pharmacokinetics and Clinical Pharmacy, Faculty of Pharmacy, University of Belgrade, 11000 Belgrade, Serbia
2 Department of Gastroenterology and Hepatology, Zvezdara University Medical Center, 11000 Belgrade, Serbia
3 Faculty of Medicine, University of Belgrade, 11000 Belgrade, Serbia
* Correspondence: katarina.vucicevic@pharmacy.bg.ac.rs

Supplementary Materials

Table S1. All adverse events by MedDRA SOC, PT, and treatment phase, with incidence rates per 100 PYs.

MedDRA SOC/ MedDRA PT	Induction	Maintenance	Other*	Total	Incidence/ 100 PYs
**Blood and lymphatic system disorders**	**0**	**9**	**0**	**9**	1.52
Leukopenia	0	1	0	1	0.17
Lymph node pain	0	1	0	1	0.17
Lymphadenopathy	0	2	0	2	0.34
Splenomegaly	0	1	0	1	0.17
Thrombocytopenia	0	3	0	3	0.51
Thrombocytosis	0	1	0	1	0.17
**Cardiac disorders**	**1**	**5**	**0**	**6**	1.01
Palpitations	0	2	0	2	0.34
Tachycardia	1	3	0	4	0.68
**Ear and labyrinth disorders**	**0**	**1**	**0**	**1**	0.17
Ear pain	0	1	0	1	0.17
**Eye disorders**	**1**	**5**	**0**	**6**	1.01
Chalazion	0	1	0	1	0.17
Conjunctivitis allergic	0	1	0	1	0.17
Eye irritation	1	0	0	1	0.17
Eye pruritus	0	1	0	1	0.17
Iridocyclitis	0	1	0	1	0.17
Vision blurred	0	1	0	1	0.17
**Gastrointestinal disorders**	**0**	**13**	**0**	**13**	2.20
Abdominal pain upper	0	1	0	1	0.17
Dysphagia	0	1	0	1	0.17
Gastrooesophageal reflux disease	0	1	0	1	0.17
Mouth swelling	0	1	0	1	0.17

Nausea	0	1	0	1	0.17
Noninfective sialoadenitis	0	1	0	1	0.17
Obstructive pancreatitis	0	2	0	2	0.34
Pancreatitis acute	0	1	0	1	0.17
Paraesthesia oral	0	1	0	1	0.17
Tongue discomfort	0	1	0	1	0.17
Vomiting	0	2	0	2	0.34
**General disorders and administration site conditions**	**3**	**52**	**0**	**55**	9.30
Asthenia	0	4	0	4	0.68
Chest discomfort	0	1	0	1	0.17
Chest pain	0	1	0	1	0.17
Chills	1	1	0	2	0.34
Drug intolerance	0	1	0	1	0.17
Fatigue	0	12	0	12	2.03
Injection site erythema	0	2	0	2	0.34
Injection site hypersensitivity	0	1	0	1	0.17
Injection site induration	0	1	0	1	0.17
Injection site reaction	0	2	0	2	0.34
Malaise	1	9	0	10	1.69
Non-cardiac chest pain	0	1	0	1	0.17
Oedema peripheral	0	1	0	1	0.17
Peripheral swelling	0	1	0	1	0.17
Pyrexia	1	14	0	15	2.54
**Hepatobiliary disorders**	**3**	**18**	**0**	**21**	3.55
Cholelithiasis	0	10	0	10	1.69
Cholestasis	1	0	0	1	0.17
Hepatic cyst	0	1	0	1	0.17
Hepatic cytolysis	2	6	0	8	1.35
Hepatic steatosis	0	1	0	1	0.17
**Immune system disorders**	**0**	**2**	**0**	**2**	0.34
Hypersensitivity	0	2	0	2	0.34
**Infections and infestations**	**9**	**182**	**0**	**191**	32.30
Abdominal abscess	0	1	0	1	0.17
Abscess	0	1	0	1	0.17
Abscess limb	0	1	0	1	0.17
Appendicitis	0	1	0	1	0.17
Bacterial disease carrier	1	2	0	3	0.51
Bacterial sepsis	0	1	0	1	0.17
Bronchitis	0	2	0	2	0.34
Clostridium difficile infection	0	3	0	3	0.51
Conjunctivitis	0	1	0	1	0.17
COVID-19	1	59	0	60	10.15

COVID-19 pneumonia	0	2	0	2	0.34
Escherichia urinary tract infection	1	3	0	4	0.68
Gastroenteritis	0	2	0	2	0.34
Gastroenteritis viral	0	5	0	5	0.85
Helicobacter infection	0	1	0	1	0.17
Herpes simplex	2	4	0	6	1.01
Herpes virus infection	0	1	0	1	0.17
Herpes zoster	1	7	0	8	1.35
Herpes zoster reactivation	0	1	0	1	0.17
Influenza	0	2	0	2	0.34
Nasal herpes	0	1	0	1	0.17
Nasopharyngitis	2	27	0	29	4.90
Oral herpes	0	5	0	5	0.85
Oral infection	0	1	0	1	0.17
Pelvic abscess	0	2	0	2	0.34
Pharyngitis	0	6	0	6	1.01
Pilonidal disease	0	1	0	1	0,17
Pneumonia	0	3	0	3	0.51
Respiratory tract infection	1	14	0	15	2.54
Rhinitis	0	2	0	2	0.34
Sinusitis	0	4	0	4	0.68
Staphylococcal skin infection	0	1	0	1	0.17
Stoma site infection	0	1	0	1	0.17
Tonsillitis	0	2	0	2	0.34
Tonsillitis bacterial	0	2	0	2	0.34
Upper respiratory tract infection	0	1	0	1	0.17
Urinary tract infection	0	6	0	6	1.01
Urinary tract infection bacterial	0	1	0	1	0.17
Urinary tract infection enterococcal	0	1	0	1	0.17
Vaginal infection	0	1	0	1	0.17
**Injury, poisoning and procedural complications**	**0**	**8**	**0**	**8**	1.35
Forearm fracture	0	1	0	1	0.17
Ligament injury	0	1	0	1	0.17
Ligament rupture	0	1	0	1	0.17
Limb injury	0	1	0	1	0.17
Meniscus injury	0	1	0	1	0.17
Stoma site rash	0	1	0	1	0.17
Upper limb fracture	0	2	0	2	0.34
**Investigations**	**6**	**95**	**0**	**101**	17.08
Alanine aminotransferase increased	0	14	0	14	2.37
Amylase increased	1	4	0	5	0.85
Antineutrophil cytoplasmic antibody increased	0	1	0	1	0.17

Antinuclear antibody increased	0	4	0	4	0.68
Aspartate aminotransferase increased	0	8	0	8	1.35
Bilirubin conjugated increased	0	1	0	1	0.17
Blood alkaline phosphatase increased	0	3	0	3	0.51
Blood bilirubin increased	1	2	0	3	0.51
Blood cholesterol increased	0	6	0	6	1.01
Blood creatine phosphokinase MB increased	0	1	0	1	0.17
Blood creatinine increased	0	3	0	3	0.51
Blood lactate dehydrogenase increased	0	2	0	2	0.34
Blood pressure increased	0	5	0	5	0.85
Blood triglycerides increased	0	7	0	7	1.18
Blood urea increased	0	2	0	2	0.34
Enterococcus test positive	0	1	0	1	0.17
Escherichia test positive	0	2	0	2	0.34
Gamma-glutamyltransferase increased	1	10	0	11	1.86
Glucose urine present	1	0	0	1	0.17
Haemophilus test positive	0	2	0	2	0.34
Hepatic enzyme increased	0	1	0	1	0.17
Lipase increased	0	4	0	4	0.68
Lipids increased	0	2	0	2	0.34
Moraxella test positive	1	0	0	1	0.17
Pancreatic enzymes increased	0	1	0	1	0.17
Streptococcus test positive	0	2	0	2	0.34
Transaminases increased	0	3	0	3	0.51
Urine ketone body present	1	0	0	1	0.17
Vitamin D decreased	0	1	0	1	0.17
Weight decreased	0	2	0	2	0.34
Weight increased	0	1	0	1	0.17
**Metabolism and nutrition disorders**	**3**	**24**	**0**	**27**	4.57
Decreased appetite	1	1	0	2	0.34
Dehydration	0	1	0	1	0.17
Dyslipidaemia	1	0	0	1	0.17
Hypercholesterolaemia	0	3	0	3	0.51
Hyperglycaemia	1	1	0	2	0.34
Hyperlipidaemia	0	6	0	6	1.01
Hypertriglyceridaemia	0	1	0	1	0.17
Hypoalbuminaemia	0	2	0	2	0.34
Hypochloraemia	0	1	0	1	0.17
Hypokalaemia	0	2	0	2	0.34
Hypomagnesaemia	0	1	0	1	0.17
Hyponatraemia	0	1	0	1	0.17
Hypophosphataemia	0	1	0	1	0.17

Hypoproteinaemia	0	1	0	1	0.17
Insulin resistance	0	1	0	1	0.17
Metabolic syndrome	0	1	0	1	0.17
**Musculoskeletal and connective tissue disorders**	**3**	**20**	**0**	**23**	3.89
Back pain	0	1	0	1	0.17
Chest wall mass	0	1	0	1	0.17
Limb mass	0	1	0	1	0.17
Lupus-like syndrome	0	3	0	3	0.51
Muscle spasms	0	5	0	5	0.85
Muscular weakness	0	1	0	1	0.17
Myalgia	1	4	0	5	0.85
Neck mass	0	1	0	1	0.17
Neck pain	1	0	0	1	0.17
Osteonecrosis	0	1	0	1	0.17
Pain in extremity	1	0	0	1	0.17
Spinal pain	0	1	0	1	0.17
Systemic lupus erythematosus	0	1	0	1	0.17
**Neoplasms benign, malignant and unspecified (incl cysts and polyps)**	**0**	**9**	**2**	**11**	1.86
Adenocarcinoma pancreas	0	0	1	1	0.17
Adrenal adenoma	0	2	0	2	0.34
Brain neoplasm	0	1	0	1	0.17
Dysplastic naevus	0	1	0	1	0.17
Fibroma	0	1	0	1	0.17
Haemangioma of liver	0	1	0	1	0.17
Hepatic cancer	0	0	1	1	0.17
Meningioma benign	0	1	0	1	0.17
Ovarian cancer	0	1	0	1	0.17
Skin papilloma	0	1	0	1	0.17
**Nervous system disorders**	**1**	**27**	**0**	**28**	4.74
Ageusia	0	1	0	1	0.17
Anosmia	0	1	0	1	0.17
Balance disorder	0	2	0	2	0.34
Demyelination	0	1	0	1	0.17
Disturbance in attention	0	1	0	1	0.17
Dizziness	0	1	0	1	0.17
Dyskinesia	0	1	0	1	0.17
Epilepsy	0	1	0	1	0.17
Headache	1	8	0	9	1.52
Hyporeflexia	0	1	0	1	0.17
Ischaemic cerebral infarction	0	2	0	2	0.34
Memory impairment	0	2	0	2	0.34
Migraine	0	1	0	1	0.17

Paraesthesia	0	3	0	3	0.51
Speech disorder	0	1	0	1	0.17
**Psychiatric disorders**	**0**	**5**	**0**	**5**	0.85
Bradyphrenia	0	1	0	1	0.17
Depressed mood	0	1	0	1	0.17
Depression	0	1	0	1	0.17
Disorganised speech	0	1	0	1	0.17
Insomnia	0	1	0	1	0.17
**Renal and urinary disorders**	**0**	**15**	**0**	**15**	2.54
Acute kidney injury	0	1	0	1	0.17
Dysuria	0	1	0	1	0.17
Nephrolithiasis	0	4	0	4	0.68
Pollakiuria	0	1	0	1	0.17
Renal colic	0	2	0	2	0.34
Renal cyst	0	4	0	4	0.68
Renal pain	0	1	0	1	0.17
Urinary incontinence	0	1	0	1	0.17
**Reproductive system and breast disorders**	**0**	**17**	**0**	**17**	2.88
Breast disorder	0	1	0	1	0.17
Breast mass	0	1	0	1	0.17
Cervical dysplasia	0	1	0	1	0.17
Cervix disorder	0	1	0	1	0.17
Heavy menstrual bleeding	0	2	0	2	0.34
Menometrorrhagia	0	1	0	1	0.17
Menstruation irregular	0	1	0	1	0.17
Ovarian cyst	0	5	0	5	0.85
Ovarian cyst ruptured	0	1	0	1	0.17
Uterine cervical metaplasia	0	1	0	1	0.17
Uterine polyp	0	2	0	2	0.34
**Respiratory, thoracic and mediastinal disorders**	**3**	**31**	**0**	**34**	5.75
Cough	0	7	0	7	1.18
Dyspnoea	0	3	0	3	0.51
Epistaxis	0	2	0	2	0.34
Nasal congestion	1	0	0	1	0.17
Nasal polyps	0	1	0	1	0.17
Oropharyngeal pain	0	6	0	6	1.01
Pneumothorax	0	1	0	1	0.17
Productive cough	0	3	0	3	0.51
Pulmonary embolism	0	1	0	1	0.17
Respiration abnormal	0	1	0	1	0.17
Rhinorrhoea	0	2	0	2	0.34
Sneezing	1	2	0	3	0.51

Throat irritation	1	0	0	1	0.17
Throat tightness	0	1	0	1	0.17
Tonsillar hypertrophy	0	1	0	1	0.17
**Skin and subcutaneous tissue disorders**	**2**	**48**	**0**	**50**	8.46
Acne	0	2	0	2	0.34
Alopecia	0	5	0	5	0.85
Dermatitis	0	2	0	2	0.34
Dermatitis psoriasiform	0	1	0	1	0.17
Eczema	0	1	0	1	0.17
Erythema	0	6	0	6	1.01
Hyperhidrosis	0	2	0	2	0.34
Nail disorder	0	1	0	1	0.17
Pain of skin	0	1	0	1	0.17
Pigmentation disorder	0	1	0	1	0.17
Pruritus	1	6	0	7	1.18
Rash	0	4	0	4	0.68
Rash macular	0	1	0	1	0.17
Rash maculo-papular	0	1	0	1	0.17
Rash papular	0	1	0	1	0.17
Rash pruritic	1	2	0	3	0.51
Rash vesicular	0	1	0	1	0.17
Skin discharge	0	1	0	1	0.17
Skin disorder	0	5	0	5	0.85
Skin exfoliation	0	1	0	1	0.17
Skin hyperpigmentation	0	1	0	1	0.17
Skin induration	0	1	0	1	0.17
Urticaria	0	1	0	1	0.17
**Vascular disorders**	**0**	**7**	**0**	**7**	1.18
Aortic occlusion	0	1	0	1	0.17
Haematoma	0	1	0	1	0.17
Hypertension	0	3	0	3	0.51
Iliac artery occlusion	0	1	0	1	0.17
Raynaud's phenomenon	0	1	0	1	0.17
**Total**	**35**	**593**	**2**	**630**	**106.55**

MedDRA: Medical Dictionary for Regulatory Activities; PT: preferred term; PYs: patient-years; SOC: system organ class.
* The “Other” category includes events not occurring during the induction or maintenance phases; specifically, two malignancies detected after adalimumab discontinuation, in accordance with the study methodology.

Table S2. New EIMs by MedDRA SOC and PT, with incidence, disproportionality analysis of FAERS data, and listedness assessment.

Study data			FAERS data				
**MedDRA SOC/ MedDRA PT**	**n**	**Incidence/ 100 PYs**	**N**	**ROR (95% CI)**	**PRR (95% CI)**	**Chi square**	**Listedness per USPI**
**Blood and lymphatic system disorders**	**33**	5.58					
Anaemia	19	3.21	1638	0.709 (0.675, 0.744)	0.71 (0.676, 0.745)	191.709	Listed
Iron deficiency anaemia	8	1.35	146	1.532 (1.298, 1.807)*	1.531 (1.298, 1.806)	25.427	Listed
Normocytic anaemia	6	1.01	11	0.576 (0.318, 1.045)	0.576 (0.318, 1.045)	2.962	Listed
**Eye disorders**	**2**	0.34					
Iridocyclitis**	1	0.17	128	2.873 (2.401, 3.437)*	2.872 (2.401, 3.436)*	144.169	Unlisted
Uveitis**	1	0.17	1092	6.392 (5.996, 6.813)*	6.373 (5.98, 6.792)*	4,278.229	Unlisted
**Musculoskeletal and connective tissue disorders**	**64**	10.82					
Arthralgia**	51	8.63	1313 7	2.57 (2.524, 2.616)*	2.506 (2.463, 2.55)*	11,395.938	Unlisted
Back pain	3	0.51	3967	1.37 (1.327, 1.414)*	1.365 (1.323, 1.409)	377.737	Unlisted
Bone pain	1	0.17	565	0.722 (0.665, 0.785)	0.723 (0.665, 0.785)	58.858	Unlisted
Joint stiffness**	2	0.34	1106	3.626 (3.409, 3.856)*	3.617 (3.401, 3.846)*	1,923.315	Unlisted
Joint swelling**	1	0.17	3570	2.413 (2.333, 2.497)*	2.398 (2.319, 2.480)*	2,759.806	Unlisted
Musculoskeletal stiffness**	1	0.17	2556	2.347 (2.255, 2.443)*	2.336 (2.245, 2.431)*	1,853.308	Unlisted
Myalgia	1	0.17	1538	0.666 (0.633, 0.700)	0.667 (0.634, 0.702)	252.695	Unlisted
Osteoporosis	1	0.17	578	0.999 (0.920, 1.085)	0.999 (0.920, 1.085)	0	Unlisted
Sacral pain	1	0.17	0	0	0		Unlisted
Spinal pain**	2	0.34	278	2.055 (1.822, 2.318)*	2.054 (1.821, 2.317)*	142.207	Unlisted
**Nervous system disorders**	**3**	0.51					
Hypoaesthesia	1	0.17	2765	1.428 (1.375, 1.483)*	1.424 (1.372, 1.479)	339.412	Unlisted
Paraesthesia	1	0.17	2334	1.114 (1.069, 1.161)*	1.114 (1.069, 1.160)	26.416	Listed
Radiculopathy**	1	0.17	95	2.874 (2.334, 3.539)*	2.874 (2.334, 3.538)*	106.657	Unlisted
**Skin and subcutaneous tissue disorders**	**9**	1.52					
Eczema	1	0.17	565	0.797 (0.734, 0.867)	0.798 (0.734, 0.867)	28.321	Unlisted
Erythema nodosum**	2	0.34	139	3.213 (2.703, 3.819)*	3.212 (2.703, 3.817)*	194.273	Unlisted
Psoriasis**	5	0.85	8492	5.144 (5.028, 5.263)*	5.036 (4.925, 5.149)*	2,4593.684	Listed
Pyoderma gangrenosum**	1	0.17	517	13.940 (12.616, 15.404)*	13.92 (12.599, 15.380)*	4,617.014	Unlisted
**Total**	**111**	18.77					

CI: confidence interval; EIMs: extraintestinal manifestations; FAERS: FDA Adverse Event Reporting System; MedDRA: Medical Dictionary for Regulatory Activities; PT: preferred term; PYs: patient-years; ROR: reporting odds ratio; PRR: proportional reporting ratio; SOC: system organ class; USPI: U.S. Prescribing Information.
* Indicates that either ROR or PRR exceeds the predefined statistical threshold; ** Indicates MedDRA PTs meeting safety signal criteria, defined as both ROR and PRR exceeding the statistical thresholds.

Table S3. Disproportionality analysis for selected adverse events and their listedness according to the adalimumab USPI.

MedDRA SOC/ MedDRA PT	N	ROR (95% CI)	PRR (95% CI)	Chi square	Listedness per USPI
**Blood and lymphatic system disorders**					
Leukopenia	173	0.274 (0.236, 0.318)	0.274 (0.236, 0.319)	329.487	Listed
Lymph node pain**	53	2.448 (1.856, 3.230)*	2.448 (1.856, 3.230)*	41.451	Unlisted
Lymphadenopathy**	976	2.292 (2.149, 2.445)*	2.289 (2.146, 2.441)*	670.281	Unlisted
Splenomegaly	133	0.894 (0.753, 1.061)	0.894 (0.753, 1.061)	1.540	Unlisted
Thrombocytopenia	226	0.156 (0.137, 0.178)	0.157 (0.138, 0.179)	1,023.654	Listed
Thrombocytosis	50	0.925 (0.699, 1.225)	0.925 (0.699, 1.225)	0.223	Unlisted
**Cardiac disorders**					
Palpitations	699	0.436 (0.405, 0.470)	0.437 (0.406, 0.471)	502.535	Listed
Tachycardia	329	0.284 (0.255, 0.316)	0.285 (0.255, 0.317)	589.260	Listed
**Ear and labyrinth disorders**					
Ear pain	425	1.849 (1.678, 2.038)*	1.848 (1.677, 2.037)	157.620	Unlisted
**Eye disorders**					
Chalazion	9	1.140 (0.588, 2.212)	1.140 (0.588, 2.212)	0.044	Unlisted
Conjunctivitis allergic	6	0.333 (0.149, 0.743)	0.333 (0.149, 0.743)	7.301	Unlisted
Eye irritation	225	0.285 (0.250, 0.325)	0.286 (0.251, 0.326)	398.813	Unlisted
Eye pruritus	184	0.378 (0.327, 0.437)	0.379 (0.327, 0.438)	185.659	Unlisted
Iridocyclitis**	128	2.873 (2.401, 3.437)*	2.872 (2.401, 3.436)*	144.169	Unlisted
Vision blurred	1218	0.642 (0.606, 0.679)	0.643 (0.608, 0.681)	238.367	Unlisted
**Gastrointestinal disorders**					
Abdominal pain upper	2373	0.908 (0.871, 0.945)	0.910 (0.874, 0.948)	21.610	Listed
Dysphagia	586	0.422 (0.389, 0.457)	0.423 (0.390, 0.458)	458.806	Unlisted
Gastrooesophageal reflux disease	647	0.627 (0.580, 0.678)	0.628 (0.581, 0.679)	140.540	Unlisted
Mouth swelling	37	0.450 (0.326, 0.622)	0.450 (0.326, 0.622)	24.036	Listed
Nausea	6562	0.603 (0.588, 0.618)	0.611 (0.597, 0.626)	1,653.764	Listed
Noninfective sialoadenitis	3	1.116 (0.354, 3.514)	1.116 (0.354, 3.514)	0.014	Unlisted
Obstructive pancreatitis	18	1.934 (1.205, 3.102)*	1.933 (1.205, 3.102)	6.860	Unlisted
Pancreatitis acute	95	0.304 (0.249, 0.372)	0.305 (0.249, 0.373)	149.189	Listed
Paraesthesia oral	138	0.733 (0.619, 0.867)	0.733 (0.619, 0.867)	12.927	Unlisted
Tongue discomfort	28	0.452 (0.311, 0.655)	0.452 (0.311, 0.655)	17.887	Unlisted
Vomiting	3806	0.606 (0.587, 0.626)	0.611 (0.591, 0.630)	949.082	Listed
**General disorders and administration site conditions**					
Asthenia	3557	0.748 (0.723, 0.773)	0.751 (0.726, 0.776)	293.597	Unlisted
Chest discomfort	775	0.595 (0.554, 0.639)	0.596 (0.555, 0.639)	210.018	Listed
Chest pain	1870	0.814 (0.777, 0.852)	0.815 (0.779, 0.853)	77.400	Listed

Chills	1508	1.043 (0.991, 1.098)	1.043 (0.991, 1.098)	2.570	Listed
Drug intolerance	1024	0.718 (0.675, 0.764)	0.719 (0.676, 0.765)	110.816	Listed
Fatigue	10652	1.010 (0.990, 1.029)	1.009 (0.990, 1.028)	0.891	Unlisted
Injection site erythema**	6579	3.894 (3.796, 3.994)*	3.835 (3.740, 3.932)*	12,676.179	Listed
Injection site hypersensitivity**	127	2.972 (2.482, 3.559)*	2.972 (2.482, 3.558)*	153.036	Listed
Injection site induration**	369	2.032 (1.830, 2.256)*	2.031 (1.829, 2.254)*	182.978	Unlisted
Injection site reaction**	3624	3.731 (3.606, 3.861)*	3.701 (3.578, 3.828)*	6,569.315	Listed
Malaise	7305	1.188 (1.160, 1.216)*	1.183 (1.157, 1.211)	205.673	Unlisted
Non-cardiac chest pain	19	0.580 (0.369, 0.913)	0.580 (0.369, 0.913)	5.272	Unlisted
Oedema peripheral	1841	1.244 (1.188, 1.303)*	1.243 (1.187, 1.302)	84.946	Listed
Peripheral swelling	2648	1.383 (1.330, 1.438)*	1.380 (1.328, 1.434)	269.345	Listed
Pyrexia	5464	1.320 (1.285, 1.356)*	1.315 (1.280, 1.350)	403.692	Listed
**Hepatobiliary disorders**					
Cholelithiasis	483	1.192 (1.089, 1.305)*	1.192 (1.089, 1.305)	14.360	Listed
Cholestasis	50	0.216 (0.164, 0.285)	0.216 (0.164, 0.286)	140.571	Unlisted
Hepatic cyst	54	1.457 (1.111, 1.911)*	1.457 (1.111, 1.911)	7.024	Unlisted
Hepatic cytolysis	62	0.273 (0.213, 0.350)	0.273 (0.213, 0.350)	118.55	Listed
Hepatic steatosis	349	1.651 (1.483, 1.838)*	1.650 (1.483, 1.837)	85.409	Unlisted
**Immune system disorders**					
Hypersensitivity	2143	0.854 (0.818, 0.892)	0.855 (0.819, 0.892)	51.856	Listed
**Infections and infestations**					
Abdominal abscess**	185	4.090 (3.516, 4.758)*	4.088 (3.514, 4.756)*	388.826	Unlisted
Abscess**	948	5.456 (5.098, 5.840)*	5.443 (5.086, 5.824)*	3,024.853	Unlisted
Abscess limb**	125	2.649 (2.210, 3.175)*	2.648 (2.210, 3.174)*	118.744	Unlisted
Appendicitis	176	1.471 (1.265, 1.710)*	1.471 (1.265, 1.709)	25.127	Listed
Bacterial disease carrier	9	0.962 (0.497, 1.862)	0.962 (0.497, 1.862)	0.002	Listed
Bacterial sepsis	34	0.912 (0.649, 1.281)	0.912 (0.649, 1.281)	0.202	Listed
Bronchitis**	1845	2.169 (2.070, 2.273)*	2.162 (2.064, 2.266)*	1,096.814	Listed
Clostridium difficile infection	446	1.855 (1.687, 2.040)*	1.854 (1.686, 2.038)	167.128	Unlisted
Conjunctivitis	214	0.827 (0.723, 0.947)	0.827 (0.723, 0.947)	7.387	Unlisted
COVID-19	1707	1.743 (1.660, 1.829)*	1.739 (1.657, 1.825)	514.838	Unlisted
COVID-19 pneumonia	53	0.661 (0.504, 0.868)	0.661 (0.504, 0.868)	8.699	Unlisted
Escherichia urinary tract infection	43	0.968 (0.716, 1.311)	0.969 (0.716, 1.310)	0.017	Listed
Gastroenteritis	136	0.810 (0.683, 0.960)	0.810 (0.683, 0.960)	5.771	Listed
Gastroenteritis viral	350	1.540 (1.384, 1.714)*	1.540 (1.384, 1.713)	63.322	Listed
Helicobacter infection	171	1.944 (1.668, 2.267)*	1.944 (1.668, 2.266)	73.925	Unlisted
Herpes simplex	86	1.305 (1.052, 1.617)*	1.305 (1.052, 1.617)	5.627	Unlisted

Herpes virus infection	123	1.680 (1.403, 2.013)*	1.680 (1.403, 2.012)	31.873	Unlisted
Herpes zoster**	1558	2.166 (2.059, 2.280)*	2.161 (2.054, 2.274)*	923.900	Unlisted
Herpes zoster reactivation**	8	2.242 (1.101, 4.569)*	2.242 (1.101, 4.569)*	4.084	Unlisted
Influenza**	3066	2.156 (2.079, 2.236)*	2.145 (2.069, 2.224)*	1,787.494	Listed
Nasal herpes**	7	2.865 (1.331, 6.164)*	2.865 (1.331, 6.164)*	6.256	Unlisted
Nasopharyngitis**	7227	3.165 (3.090, 3.243)*	3.117 (3.044, 3.192)*	9,727.557	Listed
Oral herpes	377	1.317 (1.188, 1.459)*	1.316 (1.188, 1.458)	27.477	Unlisted
Oral infection	100	1.939 (1.586, 2.370)*	1.939 (1.586, 2.369)	42.486	Unlisted
Pelvic abscess**	32	2.373 (1.661, 3.388)*	2.372 (1.661, 3.388)*	22.705	Unlisted
Pharyngitis**	370	2.577 (2.320, 2.863)*	2.576 (2.319, 2.861)*	334.162	Listed
Pilonidal disease**	38	7.662 (5.418, 10.834)*	7.661 (5.418, 10.832)*	179.672	Unlisted
Pneumonia	4761	1.124 (1.091, 1.157)*	1.122 (1.090, 1.154)	61.975	Listed
Respiratory tract infection	507	1.598 (1.462, 1.746)*	1.597 (1.461, 1.745)	108.363	Listed
Rhinitis	154	1.767 (1.504, 2.077)*	1.767 (1.504, 2.076)	48.400	Listed
Sinusitis**	4103	3.465 (3.356, 3.578)*	3.434 (3.327, 3.545)*	6,554.415	Listed
Staphylococcal skin infection**	49	3.388 (2.531, 4.534)*	3.387 (2.531, 4.533)*	73.906	Unlisted
Stoma site infection	10	0.894 (0.478, 1.672)	0.894 (0.478, 1.672)	0.040	Unlisted
Tonsillitis**	206	3.291 (2.855, 3.793)*	3.289 (2.854, 3.791)*	301.703	Unlisted
Tonsillitis bacterial**	6	2.889 (1.262, 6.611)*	2.889 (1.262, 6.611)*	5.232	Unlisted
Upper respiratory tract infection**	1416	2.639 (2.501, 2.785)*	2.632 (2.495, 2.777)*	1,347.117	Listed
Urinary tract infection	3180	1.511 (1.458, 1.565)*	1.506 (1.454, 1.560)	523.777	Listed
Urinary tract infection bacterial	16	0.548 (0.335, 0.898)	0.548 (0.335, 0.898)	5.424	Listed
Urinary tract infection enterococcal	1	0.121 (0.017, 0.865)	0.121 (0.017, 0.865)	5.472	Listed
Vaginal infection	92	1.562 (1.268, 1.923)*	1.562 (1.268, 1.923)	17.353	Unlisted
**Injury, poisoning and procedural complications**					
Forearm fracture	12	1.479 (0.832, 2.631)	1.479 (0.832, 2.631)	1.355	Listed
Ligament injury**	43	3.520 (2.578, 4.806)*	3.520 (2.578, 4.806)*	69.062	Unlisted
Ligament rupture**	279	5.626 (4.964, 6.376)*	5.622 (4.961, 6.371)*	928.091	Unlisted
Limb injury**	729	2.173 (2.017, 2.341)*	2.170 (2.014, 2.338)*	436.088	Unlisted
Meniscus injury**	576	8.119 (7.424, 8.879)*	8.106 (7.414, 8.864)*	2,989.676	Unlisted
Stoma site rash**	3	4.911 (1.483, 16.266)*	4.911 (1.483, 16.266)*	5.149	Unlisted
Upper limb fracture	394	1.387 (1.255, 1.534)*	1.387 (1.254, 1.533)	40.768	Listed
**Investigations**					
Alanine aminotransferase increased	238	0.336 (0.296, 0.382)	0.337 (0.297, 0.383)	308.159	Listed
Amylase increased	15	0.217 (0.131, 0.361)	0.217 (0.131, 0.361)	41.257	Unlisted
Antineutrophil cytoplasmic antibody increased	3	1.395 (0.441, 4.409)	1.395 (0.441, 4.409)	0.051	Listed
Antinuclear antibody increased**	80	7.853 (6.182, 9.976)*	7.851 (6.181, 9.973)*	395.452	Listed

Aspartate aminotransferase increased	175	0.297 (0.256, 0.344)	0.297 (0.256, 0.345)	288.592	Listed
Bilirubin conjugated increased	2	0.077 (0.019, 0.310)	0.077 (0.019 , 0.310)	21.034	Unlisted
Blood alkaline phosphatase increased	51	0.191 (0.145, 0.252)	0.191 (0.145, 0.252)	172.894	Listed
Blood bilirubin increased	68	0.210 (0.165, 0.266)	0.210 (0.166, 0.267)	200.266	Unlisted
Blood cholesterol increased	499	0.882 (0.807, 0.963)	0.882 (0.807, 0.964)	7.635	Listed
Blood creatine phosphokinase MB increased	1	0.084 (0.012, 0.600)	0.084 (0.012, 0.600)	9.001	Unlisted
Blood creatinine increased	214	0.261 (0.228, 0.298)	0.261 (0.228, 0.299)	445.104	Unlisted
Blood lactate dehydrogenase increased	30	0.189 (0.132, 0.271)	0.189 (0.132, 0.271)	102.885	Unlisted
Blood pressure increased	823	0.416 (0.388, 0.445)	0.417 (0.389, 0.447)	666.986	Listed
Blood triglycerides increased	129	0.568 (0.477, 0.675)	0.568 (0.477, 0.675)	41.470	Listed
Blood urea increased	38	0.221 (0.161, 0.304)	0.221 (0.161, 0.305)	102.723	Unlisted
Enterococcus test positive	2	0.262 (0.065, 1.054)	0.262 (0.065, 1.054)	3.405	Unlisted
Escherichia test positive	6	0.429 (0.192, 0.960)	0.429 (0.192, 0.960)	3.945	Unlisted
Gamma-glutamyltransferase increased	57	0.247 (0.190, 0.320)	0.247 (0.190, 0.320)	129.635	Unlisted
Glucose urine present	6	0.406 (0.182, 0.907)	0.406 (0.182, 0.907)	4.582	Unlisted
Haemophilus test positive	0	0	0	0.839	Unlisted
Hepatic enzyme increased	1184	1.373 (1.296, 1.456)*	1.372 (1.295, 1.454)	115.554	Listed
Lipase increased	42	0.431 (0.318, 0.584)	0.431 (0.318, 0.584)	30.714	Unlisted
Lipids increased	17	0.590 (0.366, 0.952)	0.590 (0.366, 0.952)	4.367	Listed
Moraxella test positive	0	0	0	0.012	Unlisted
Pancreatic enzymes increased	10	0.500 (0.268, 0.933)	0.500 (0.268, 0.933)	4.438	Unlisted
Streptococcus test positive	19	1.467 (0.928, 2.319)	1.467 (0.928, 2.319)	2.286	Unlisted
Transaminases increased	119	0.401 (0.335, 0.480)	0.401 (0.335, 0.480)	104.875	Listed
Urine ketone body present	2	0.100 (0.025, 0.400)	0.100 (0.025, 0.400)	15.267	Unlisted
Vitamin D decreased	121	1.665 (1.388, 1.997)*	1.665 (1.388, 1.996)	30.201	Unlisted
Weight decreased	3102	0.906 (0.874, 0.938)	0.906 (0.875, 0.939)	29.544	Unlisted
Weight increased	2025	0.677 (0.648, 0.708)	0.679 (0.650, 0.710)	304.355	Unlisted
**Metabolism and nutrition disorders**					
Decreased appetite	1616	0.525 (0.500, 0.551)	0.527 (0.502, 0.554)	681.71	Unlisted
Dehydration	1218	0.785 (0.742, 0.831)	0.786 (0.743, 0.832)	69.524	Listed
Dyslipidaemia	29	0.445 (0.309, 0.642	0.445 (0.309, 0.642	19.283	Listed
Hypercholesterolaemia	78	0.959 (0.766, 1.200)	0.959 (0.766, 1.200)	0.098	Listed
Hyperglycaemia	73	0.157 (0.125, 0.198)	0.158 (0.125, 0.198)	326.793	Unlisted
Hyperlipidaemia	33	0.256 (0.182, 0.361)	0.257 (0.182, 0.361)	69.944	Listed
Hypertriglyceridaemia	28	0.380 (0.262, 0.551)	0.380 (0.262, 0.551)	27.495	Listed
Hypoalbuminaemia	39	0.472 (0.344, 0.647)	0.472 (0.344, 0.647)	22.218	Unlisted
Hypochloraemia	1	0.078 (0.011, 0.552)	0.078 (0.011, 0.552)	10.012	Unlisted

Hypokalaemia	134	0.241 (0.204, 0.286)	0.242 (0.204, 0.286)	316.589	Unlisted
Hypomagnesaemia	23	0.122 (0.081, 0.184)	0.122 (0.081, 0.184)	143.499	Unlisted
Hyponatraemia	87	0.124 (0.100, 0.152)	0.124 (0.100, 0.153)	538.405	Unlisted
Hypophosphataemia	11	0.124 (0.069, 0.225)	0.124 (0.069, 0.225)	66.816	Unlisted
Hypoproteinaemia	8	0.408 (0.203, 0.818)	0.408 (0.203, 0.818)	6.228	Unlisted
Insulin resistance	6	0.203 (0.091, 0.452)	0.203 (0.091, 0.452)	17.912	Unlisted
Metabolic syndrome	14	0.531 (0.313, 0.900)	0.531 (0.313, 0.900)	5.261	Unlisted
**Musculoskeletal and connective tissue disorders**					
Back pain	3967	1.370 (1.327, 1.414)*	1.365 (1.323, 1.409)	377.737	Listed
Chest wall mass	5	1.373 (0.563, 3.348)	1.373 (0.563, 3.348)	0.191	Unlisted
Limb mass**	131	3.641 (3.045, 4.353)*	3.640 (3.044, 4.352)*	227.852	Unlisted
Lupus-like syndrome**	722	7.480 (6.909, 8.098)*	7.465 (6.897, 8.081)*	3,414.581	Listed
Muscle spasms	1863	0.767 (0.732, 0.803)	0.768 (0.734, 0.804)	128.767	Listed
Muscular weakness	1159	0.789 (0.744, 0.836)	0.790 (0.745, 0.837)	63.87	Unlisted
Myalgia	1538	0.666 (0.633, 0.700)	0.667 (0.634, 0.702)	252.695	Unlisted
Neck mass**	112	3.303 (2.725, 4.005)*	3.302 (2.724, 4.003)*	164.193	Unlisted
Neck pain	1068	1.576 (1.483, 1.676)*	1.574 (1.481, 1.674)	215.466	Unlisted
Osteonecrosis	162	0.305 (0.261, 0.355)	0.305 (0.261, 0.356)	254.514	Listed
Pain in extremity	6361	1.742 (1.698, 1.786)*	1.727 (1.685, 1.770)	1,889.092	Listed
Spinal pain**	278	2.055 (1.822, 2.318)*	2.054 (1.821, 2.317)*	142.207	Unlisted
Systemic lupus erythematosus**	2357	6.218 (5.953, 6.494)*	6.180 (5.919, 6.452)*	8,897.756	Unlisted
**Neoplasms benign, malignant and unspecified (incl cysts and polyps)**					
Adenocarcinoma pancreas	9	0.600 (0.311, 1.158)	0.606 (0.314, 1.170)	1.981	Unlisted
Adrenal adenoma	9	1.306 (0.673, 2.536)	1.306 (0.673, 2.536)	0.359	Listed
Brain neoplasm**	301	2.130 (1.897 , 2.392)*	2.129 (1.896, 2.390)*	170.277	Unlisted
Dysplastic naevus**	17	4.117 (2.500, 6.779)*	4.117 (2.500, 6.778)*	33.604	Unlisted
Fibroma**	22	3.021 (1.960, 4.659)*	3.021 (1.959, 4.659)*	25.811	Unlisted
Haemangioma of liver	19	1.662 (1.050, 2.629)*	1.661 (1.050, 2.628)	4,18	Unlisted
Hepatic cancer	120	0.222 (0.186, 0.266)	0.222 (0.186, 0.266)	324.537	Unlisted
Meningioma benign	10	1.112 (0.593, 2.084)	1.112 (0.593, 2.084)	0.027	Unlisted
Ovarian cancer	104	0.690 (0.568, 0.838)	0.690 (0.569, 0.838)	13.923	Unlisted
Skin papilloma**	181	3.748 (3.219, 4.365)*	3.747 (3.218, 4.363)*	331.387	Unlisted
**Nervous system disorders**					
Ageusia	124	0.424 (0.355, 0.506)	0.424 (0.355, 0.506)	95.603	Unlisted
Anosmia	87	0.659 (0.533, 0.814)	0.659 (0.533, 0.814)	14.815	Unlisted
Balance disorder	494	0.435 (0.398, 0.475)	0.435 (0.398, 0.476)	358.593	Unlisted
Disturbance in attention	171	0.234 (0.201, 0.271)	0.234 (0.201, 0.272)	426.608	Unlisted

Dizziness	4133	0.606 (0.588, 0.625)	0.611 (0.593, 0.630)	1,029.476	Unlisted
Dyskinesia	101	0.179 (0.147, 0.217)	0.179 (0.147, 0.217)	379.004	Unlisted
Epilepsy	321	0.758 (0.678, 0.846)	0.758 (0.679, 0.846)	24.159	Unlisted
Headache	8258	0.975 (0.953, 0.996)	0.975 (0.954, 0.997)	5.165	Listed
Hyporeflexia	9	0.377 (0.196, 0.727)	0.377 (0.196, 0.727)	8,54	Unlisted
Ischaemic cerebral infarction	10	0.723 (0.387, 1.351)	0.723 (0.387, 1.351)	0.782	Listed
Memory impairment	1128	0.563 (0.531, 0.598)	0.565 (0.533, 0.599)	374.592	Unlisted
Migraine	1472	1.156 (1.097, 1.217)*	1.155 (1.097, 1.216)	29.806	Unlisted
Paraesthesia	2334	1.114 (1.069, 1.161)*	1.114 (1.069, 1.160)	26.416	Listed
Speech disorder	347	0.481 (0.433, 0.535)	0.482 (0.433, 0.535)	191.179	Unlisted
**Nervous system disorders**					
Demyelination**	318	6.676 (5.930, 7.517)*	6.670 (5.925, 7.51)*	1,313.283	Listed
**Psychiatric disorders**					
Bradyphrenia	17	0.172 (0.107, 0.277)	0.172 (0.107, 0.277)	66.563	Unlisted
Depressed mood	496	0.661 (0.605, 0.722)	0.661 (0.605, 0.723)	84.565	Unlisted
Depression	2088	0.654 (0.626, 0.683)	0.656 (0.629, 0.685)	372.849	Unlisted
Disorganised speech	4	0.137 (0.051, 0.366)	0.137 (0.051, 0.366)	20.781	Unlisted
Insomnia	1836	0.505 (0.482, 0.529)	0.508 (0.485, 0.531)	875.641	Unlisted
**Renal and urinary disorders**					
Acute kidney injury	494	0.185 (0.170, 0.202)	0.187 (0.171, 0.204)	1,757.839	Unlisted
Dysuria	402	0.877 (0.794, 0.968)	0.877 (0.794, 0.968)	6.702	Unlisted
Nephrolithiasis	1118	1.906 (1.795, 2.024)*	1.903 (1.792, 2.02)	457.551	Listed
Pollakiuria	313	0.572 (0.512, 0.640)	0.572 (0.512, 0.640)	98.325	Unlisted
Renal colic**	76	2.954 (2.341, 3.729)*	2.954 (2.340, 3.728)*	89.753	Unlisted
Renal cyst	135	1.561 (1.314, 1.854)*	1.561 (1.314, 1.853)	25.651	Unlisted
Renal pain	109	0.777 (0.643, 0.94)	0.778 (0.643, 0.940)	6.588	Unlisted
Urinary incontinence	200	0.535 (0.465, 0.615)	0.535 (0.466, 0.615)	79.288	Unlisted
**Reproductive system and breast disorders**					
Breast disorder	42	1.442 (1.060, 1.962)*	1.442 (1.060, 1.962)	5.068	Unlisted
Breast mass**	269	3.277 (2.894, 3.711)*	3.275 (2.893, 3.708)*	391.661	Unlisted
Cervical dysplasia**	94	3.355 (2.718, 4.140)*	3.354 (2.718, 4.139)*	141.325	Unlisted
Cervix disorder	14	1.401 (0.822, 2.386)	1.401 (0.822, 2.386)	1.181	Unlisted
Heavy menstrual bleeding	205	0.566 (0.493, 0.649)	0.566 (0.493, 0.649)	66.998	Unlisted
Menometrorrhagia	7	0.271 (0.129, 0.570)	0.271 (0.129, 0.570)	12.904	Unlisted
Menstruation irregular	136	0.329 (0.278, 0.389)	0.329 (0.278, 0.390)	184.112	Listed
Ovarian cyst**	331	2.823 (2.525, 3.156)*	2.821 (2.524, 3.153)*	362.372	Unlisted
Ovarian cyst ruptured**	47	2.875 (2.139, 3.865)*	2.875 (2.139, 3.865)*	51.929	Unlisted

Uterine cervical metaplasia	2	2.728 (0.652, 11.417)	2.728 (0.652, 11.417)*	0.728	Unlisted
Uterine polyp**	79	4.067 (3.228, 5.126)*	4.067 (3.227, 5.125)*	163.35	Unlisted
**Respiratory, thoracic and mediastinal disorders**					
Cough	4534	1.266 (1.229, 1.305)*	1.263 (1.226, 1.300)	242.639	Listed
Dyspnoea	3972	0.533 (0.517, 0.550)	0.539 (0.522, 0.556)	1,582.797	Listed
Epistaxis	523	0.534 (0.490, 0.582)	0.535 (0.491, 0.583)	209.199	Unlisted
Nasal congestion	1087	1.427 (1.343, 1.516)*	1.426 (1.342, 1.515)	133.473	Unlisted
Nasal polyps	59	0.906 (0.700, 1.173)	0.906 (0.700, 1.173)	0.468	Unlisted
Oropharyngeal pain**	2387	2.023 (1.941, 2.108)*	2.015 (1.934, 2.099)*	1,166.611	Unlisted
Pneumothorax	292	1.393 (1.239, 1.565)*	1.392 (1.239, 1.564)	30.847	Unlisted
Productive cough	594	1.101 (1.014, 1.194)*	1.101 (1.014, 1.194)	5.220	Unlisted
Pulmonary embolism	610	0.469 (0.433, 0.508)	0.470 (0.434, 0.509)	360.709	Listed
Respiration abnormal	32	0.329 (0.233, 0.466)	0.329 (0.233, 0.466)	42.678	Unlisted
Rhinorrhoea	1208	1.426 (1.346, 1.510)*	1.424 (1.345, 1.508)	147.522	Unlisted
Sneezing	335	1.116 (1.001, 1.244)*	1.116 (1.001, 1.244)	3.814	Unlisted
Throat irritation	445	0.758 (0.690, 0.832)	0.758 (0.690, 0.832)	33.585	Unlisted
Throat tightness	174	0.489 (0.421, 0.568)	0.489 (0.422, 0.568)	91.137	Unlisted
Tonsillar hypertrophy**	80	3.760 (2.990, 4.727)*	3.759 (2.990, 4.726)*	145.800	Unlisted
**Skin and subcutaneous tissue disorders**					
Acne	730	1.523 (1.414, 1.640)*	1.521 (1.413, 1.638)	125.37	Unlisted
Alopecia	2764	1.030 (0.992, 1.069)	1.030 (0.992, 1.070)	2.411	Listed
Dermatitis	231	0.904 (0.793, 1.030)	0.904 (0.793, 1.030)	2.208	Unlisted
Dermatitis psoriasiform**	135	5.527 (4.617, 6.615)*	5.525 (4.616 , 6.612)*	436.747	Unlisted
Eczema	565	0.797 (0.734, 0.867)	0.798 (0.734, 0.867)	28.321	Unlisted
Erythema	2273	1.028 (0.986, 1.071)	1.027 (0.986, 1.071)	1.599	Unlisted
Hyperhidrosis	1163	0.688 (0.649, 0.729)	0.689 (0.650, 0.730)	161.186	Unlisted
Nail disorder	128	1.291 (1.083, 1.539)*	1.291 (1.083, 1.539)	7.849	Unlisted
Pain of skin	358	1.906 (1.715, 2.120)*	1.905 (1.714, 2.118)	146.386	Unlisted
Pigmentation disorder	51	0.594 (0.451, 0.783)	0.594 (0.451, 0.784)	13.520	Unlisted
Pruritus	4600	0.862 (0.837, 0.888)	0.864 (0.839, 0.890)	97.697	Unlisted
Rash	6915	1.122 (1.095, 1.149)*	1.119 (1.093, 1.146)	87.173	Listed
Rash macular**	816	2.063 (1.922, 2.213)*	2.060 (1.920, 2.210)*	423.246	Unlisted
Rash maculo-papular	99	0.352 (0.289, 0.429)	0.352 (0.289, 0.430)	116.211	Unlisted
Rash papular**	841	4.251 (3.959, 4.564)*	4.242 (3.952, 4.554)*	1,886.516	Unlisted
Rash pruritic	1130	1.661 (1.565, 1.763)*	1.659 (1.563, 1.760)	284.128	Unlisted
Rash vesicular	99	1.710 (1.399, 2.091)*	1.710 (1.399, 2.091)	27.34	Unlisted
Skin discharge**	40	9.745 (6.903, 13.758)*	9.744 (6.902, 13.756)*	246.328	Unlisted

Skin disorder**	1117	2.452 (2.309, 2.605)*	2.447 (2.304, 2.599)*	902.137	Unlisted
Skin exfoliation	957	1.121 (1.051, 1.195)*	1.121 (1.051, 1.195)	11.966	Unlisted
Skin hyperpigmentation	26	0.333 (0.226, 0.489)	0.333 (0.226, 0.489)	33.847	Unlisted
Skin induration**	73	3.431 (2.702, 4.356)*	3.430 (2.701, 4.355)*	113.676	Unlisted
Urticaria	2136	0.948 (0.908, 0.990)	0.949 (0.909, 0.990)	5.781	Listed
**Vascular disorders**					
Aortic occlusion**	14	2.879 (1.675, 4.950)*	2.879 (1.675 , 4.950)*	14.294	Unlisted
Haematoma	183	0.565 (0.489, 0.654)	0.566 (0.489, 0.654)	59.872	Unlisted
Hypertension	2052	0.763 (0.730 , 0.797)	0.764 (0.732, 0.798)	147.237	Listed
Iliac artery occlusion**	10	2.966 (1.561 , 5.635)*	2.966 (1.561, 5.635)*	10.343	Unlisted
Raynaud's phenomenon	69	1.023 (0.806, 1.299)	1.023 (0.806, 1.299)	0.016	Unlisted

CI: confidence interval; MedDRA: Medical Dictionary for Regulatory Activities; N: number of cases; PRR: proportional reporting ratio; PT: preferred term; ROR: reporting odds ratio; SOC: system organ class; USPI: U.S. Prescribing Information.

* Indicates that either ROR or PRR exceeds the predefined statistical threshold; ** Indicates MedDRA PTs meeting safety signal criteria, defined as both ROR and PRR exceeding the statistical thresholds.
